# Genetically modulating the RNA-binding protein Regnase-1 reveals its critical role in regulatory T cell homeostasis and function in vivo

**DOI:** 10.1038/s41419-026-09055-8

**Published:** 2026-07-01

**Authors:** Xiaojun Su, Xiang Xiao, Ge Deng, Si Sun, Mou Wen, Ashton A. Connor, Zhuyun Mao, Rafik M. Ghobrial, Wenhao Chen, Xian C. Li

**Affiliations:** 1 Immunobiology and Transplant Science Center, Department of Surgery, Houston Methodist Hospital, Texas Medical Center, Houston, TX USA; 2 Organ Transplant Center, The First Affiliated Hospital of Sun Yat-sen University, Guangzhou, China; 3 JC Walter Jr. Transplant Center and Conover Center for Liver Diseases and Transplantation, Houston Methodist Hospital, Houston, TX USA; 4 Department of Surgery, Weill Cornell Medical College of Cornell University, New York, NY USA

**Keywords:** Autoimmunity, Immune cell death, T cells

## Abstract

Regnase-1 is a CCCH-type RNA-binding protein that constantly degrades mRNA species via its ribonuclease domain to maintain immune quiescence. But how and by what mechanisms Regnase-1 regulates Foxp3^+^ Tregs remains poorly studied. Here, we generated a conditional knock-in strain in which a degradation-resistant Regnase-1 mutant (R111A) was selectively expressed in Foxp3^+^ Tregs as a transgene (*Foxp3*^*YFP-Cre*^*LSL-R111A*^*fl/fl*^) to investigate the roles of Regnase-1 in Tregs. We found that all male *Foxp3*^*YFP-Cre*^*LSL-R111A*^*fl/fl*^ mice died of fatal autoimmune diseases by 4 weeks of age, which was accompanied by massive activation of T effector cells in vivo. We also found that the Foxp3^+^ Tregs in male *Foxp3*^*YFP-Cre*^*LSL-R111A*^*fl/fl*^ mice were progressively depleted in the periphery. Further analyses showed that the Foxp3^+^ Tregs expressing the R111A mutant displayed an intrinsic survival defect and that wild-type Foxp3^+^ Tregs adoptively transferred into young *Foxp3*^*YFP-Cre*^*LSL-R111A*^*fl/fl*^ mice could rescue the lethal autoimmune phenotype. Mechanistically, we demonstrated that the Regnase-1 readily degraded *Bcl2l1* mRNA (encoding Bcl-xL) via binding to the *Bcl2l1* mRNA stem loop structure, thus preventing Bcl-xL expression in Tregs. Thus, Tregs expressing the R111A mutant exhibited not only survival defects but also failure to respond to IL-2, resulting in their eventual demise in vivo. Together, our studies suggest that Regnase-1 can have a profound impact on Foxp3^+^ Tregs and that modulating Regnase-1 in Tregs may have important therapeutic implications.

## Introduction

Signaling through the TCR is instrumental to the effector (suppressive) functions of both conventional (Tconv) and regulatory T cells (Tregs). One of the key events in TCR signaling is the assembly of CARMA-1, BCL10, and MALT1 signalosome, a large multi-protein multi-functional structure called the CBM complex [1]. The CBM complex acts as a signaling hub and promotes T cell activities via two major mechanisms [2]. Firstly, it functions as a scaffold, and by recruiting the E3 ubiquitin ligase TRAF6, it activates the NF-kB, MAPK, and AP-1 pathways, pathways that are central to productive T cell activation [3, 4]. Additionally, the assembly of the CBM complex also activates the paracaspase activities of MALT1, which degrades a list of protein substrates in a timely fashion (~30 substrates) [5]. As most of those substrates, including Regnase-1 (also known as Zc3h12a or MCPIP1), are potent inhibitors of cell activation, their timely degradation is also quintessential to robust T cell activation [6, 7].

As a substrate of MALT1, Regnase-1 is one of the earliest degraded molecules following TCR engagement [8]. As a ribonuclease, Regnase-1 degrades mRNAs encoding cytokines (e.g., IL-2, IL-6, IL-12), costimulatory molecules (ICOS, OX40), transcriptional regulators (c-Rel, IkBζ) and cell adhesion molecules (CD44, Tensin). As a T cell quiescent factor, the timely cleavage of Regnase-1 ensures T cell activation to proceed to becoming effector cells [7, 9]. Indeed, genetic deletion of *Zc3h12a* (encoding Regnase-1) results in massive activation of T cells, B cells, and myeloid cells, leading to spontaneous death of the Regnase-1-deleted mice by ~12 weeks of age [10]. Interestingly, this autoimmune phenotype can also be replicated in mice with conditional deletion of *Zc3h12a* specifically in T cells [11], suggesting that the lethal autoimmune phenotype of Regnase-1-deleted mice is mediated by T cells. At a cellular level, the Regnase-1-deleted T cells exhibit a prominent effector/memory phenotype, produce increased levels of IFN-γ, IL-17, and IL-4, and display a marked survival advantage in vivo [11]. activities that are consistent with an inhibitory role of Regnase-1. Moreover, the Regnase-1-deleted T cells can convert naïve T cells to an effector/memory phenotype upon transferring into the secondary hosts and show superior anti-tumor immunity [12–14]. Interestingly, initial studies suggest that in the Regnase-1-deleted mice, the Foxp3^+^ Tregs seem not altered, neither their intra-thymic development nor their suppressive activities in the periphery [10, 11].

It is puzzling that mice transgenically expressing the paracaspase-dead MALT1, which fails to degrade its substrates, also develop systemic autoimmune diseases due to unbalanced activities of T effectors and Foxp3^+^ Tregs [15]. While the T effector cells are impaired, the Foxp3^+^ Tregs are markedly reduced and functionally defective [15, 16]. In contrast, mice with an MALT1 mutant that fails to mediate its scaffold functions (with its paracaspase activities preserved) experience multi-organ inflammation as a result of hyper-activation of T effector cells [17]. The exact mechanism for such phenotypic differences remains unclear. But Foxp3^+^ Tregs are known to follow a different developmental trajectory in the thymus and phenotypically resemble effector/memory cells in the periphery [18]. Also, studies using the *Nur77-gfp* reporter mice demonstrate constitutive TCR signaling in Tregs as opposed to Tconv cells, even under homeostatic conditions [19]. which may preferentially affect the CBM complex assembly and/or activities. Thus, the role of Regnase-1 in Tregs may not be fully captured in “loss-of-function” studies. Given the importance of Tregs in the immune system and the role of Regnase-1 in CBM signaling, a detailed study of this topic is an important and clinically relevant issue.

In the present study, we generated a degradation-resistant Regnase-1 mutant R111A and expressed this mutant selectively in Foxp3^+^ Tregs as a transgene (*Foxp3*^*YFP-Cre*^*LSL-R111A*^*fl/fl*^ mice) to study the roles of Regnase-1 in Tregs. To our surprise, all the male *Foxp3*^*YFP-Cre*^*LSL-R111A*^*fl/fl*^ mice died of aggressive autoimmune diseases by 4 weeks of age because of progressive loss of Foxp3^+^ Tregs in the periphery. We provide strong evidence that Tregs that express the R111A mutant have profound survival defects and completely fail to respond to IL-2 in vivo, primarily due to the accelerated decay of *Bcl2l1* mRNA. Collectively, our data provide new insights into the role of Regnase-1 in regulating Tregs and may have important implications in manipulating Tregs for therapeutic purposes.

## Results

### Generation of GFP reporter mice transgenically expressing an MALT1-resistant Regnase-1 mutant

**Fig. 1:**
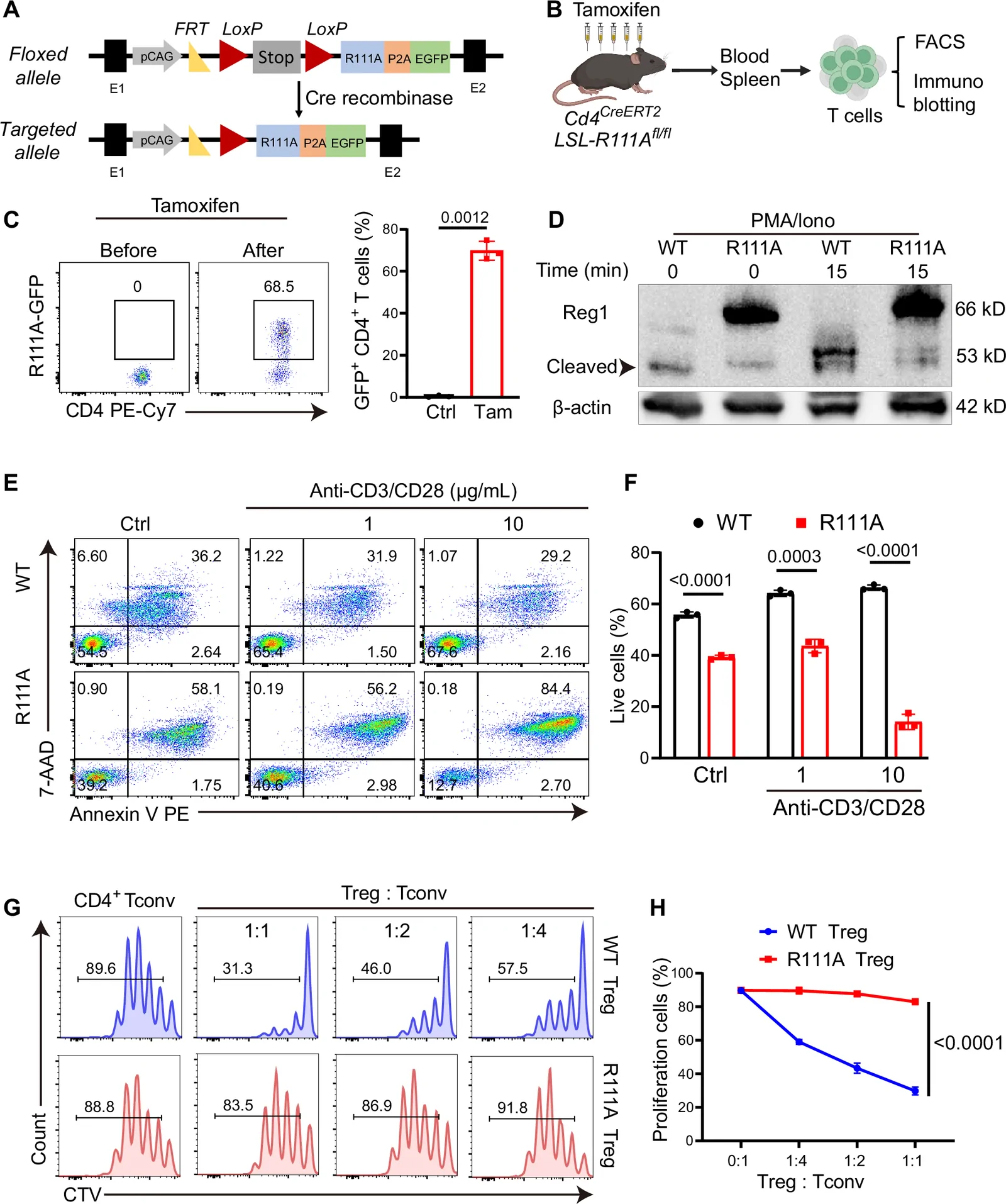
Conditional expression of the R111A mutant as a transgene impairs T cell survival and Treg functions. **A** A diagram showing targeting strategy for transgenic expression of the R111A mutant, where the R111A mutant is under the control of the floxed STOP cassette and additionally marked by EGFP, designated hereafter as *LSL-R111A*^*fl/fl*^. **B** A cartoon rendering of *Cd4*^*CreERT2*^*LSL-R111A*^*fl/fl*^ mice, in which expression of the R111A mutant in T cells is induced upon tamoxifen administration (5 doses). T cells were prepared from the spleen and blood of the treated mice for further analyses. **C** A representative flow cytometry plot (left) showing R111A-GFP expression in the blood of *Cd4*^*CreERT2*^*LSL-R111A*^*fl/fl*^ mice before and after tamoxifen treatment and the percentage of GFP^+^ cells in the CD4^+^ cell population (right, *n* = 3 replicates). **D** Immunoblot showing Regnase-1 cleavage in WT and R111A CD4^+^ T cells before and after stimulation with PMA plus ionomycin for 15 mins. β-actin was used as a loading control. FACS sorted T cells from tamoxifen-treated WT (CD4^+^CD25^-^) and *Cd4*^*CreERT2*^*LSL-R111A*^*fl/fl*^ (GFP^+^CD4^+^CD25^-^) mice were activated with different concentration of plate-coated anti-CD3 (0–10 µg/mL) and fixed dose of anti-CD28 (1 µg/mL) in vitro for 24 h, and cell survival was assessed by staining with Annexin V and 7-AAD. Representative flow cytometric plots (**E**) showing Annexin V and 7-AAD staining, and the bar graphs (**F**) showing the mean percentages of live cells in WT and R111A T cells (*n* = 3 independent replicates). In vitro CTV dilution assays comparing the suppressive functions of sorted GFP^+^CD4^+^CD25^+^ Tregs from Foxp3-GFP reporter and tamoxifen-treated *Cd4*^*CreERT2*^*LSL-R111A*^*fl/fl*^ mice. CTV-labeled conventional CD4^+^ T (Tconv, CD45.1^+^) cells were co-cultured with sorted WT or R111A Tregs (both are CD45.2^+^) at different ratios in the presence of anti-CD3 and APCs. Flow cytometry plots (**G**) show different ratios between Tregs and Tconv cells in the culture. The line graphs (**H**) show a summary of Tconv cell proliferation with or without Tregs (*n* = 3 independent replicates). All data are presented as mean ± SD. Results are representative of three independent experiments (**C**–**H**). Statistical significance was determined by two-tailed paired Student’s *t* test (**C**), two-tailed unpaired Student’s *t* test (**F**) or two-way ANOVA (**H**).

To study the role of Regnase-1 mechanistically, we generated a GFP reporter mouse strain carrying a mutant *Regnase-1* allele (Fig.1A), in which the Arginine at position 111, which is the cleavage site of the MALT1 paracaspase [11]. was replaced with an Alanine (i.e., R111A mutant). The targeting vector was flanked by an *Egfp* sequence and a Stop cassette, with 2 additional *LoxP* sites inserted flanking the Stop cassette (*LSL-R111A*^*fl/fl*^ mice). Hence, the *Cre*-mediated recombination would delete the Stop cassette, allowing expression of the R111A mutant as a transgene in targeted cells, which can be tracked by EGFP expression. We then bred the *LSL-R111A*^*fl/fl*^ mice with *Cd4*^*CreERT2*^ mice, in which expression of the R111A mutant in T cells was induced only upon tamoxifen treatment (Fig. 1B). As shown in Fig. 1C, ~70% of peripheral blood CD4^+^ T cells from *Cd4*^*CreERT2*^*LSL-R111A*^*fl/fl*^ mice became EGFP^+^ after a brief treatment with tamoxifen (75 mg/kg i.p. daily for 5 days). Moreover, stimulation of CD4^+^ T cells in vitro with PMA and ionomycin, which directly activates the CBM complex [20]. showed that the R111A mutant was highly resistant to degradation and consistently expressed at high levels regardless of PMA/ionomycin stimulation, which was in stark contrast to that in the WT control T cells (Fig. 1D).

We FACS sorted the GFP^+^ T cells from tamoxifen-treated *Cd4*^*CreERT2*^*LSL-R111A*^*fl/fl*^ mice into CD4^+^CD25^–^ (Tconv) and CD4^+^CD25^+^ (Tregs) populations and further examined their effector and suppressive activities in vitro, respectively. FACS sorted Tconv and Tregs from tamoxifen-treated WT B6 mice, and Foxp3-GFP reporter mice were included as controls. As shown in Fig. 1E, F, stimulation of the R111A Tconv with anti-CD3/CD28 induced prominent apoptotic cell death, and >80% of the Tconv cells were dead when stimulated with a higher dose of anti-CD3, as shown by Annexin V and 7-AAD staining, suggesting a substantial survival defect of R111A-expressing Tconv cells. Moreover, we co-transferred WT TEa and R111A TEa cells (TCR-transgenic CD4^+^ T cells specific for the BALB/c Eα_52-68_ alloantigen) into syngeneic *Rag1*^*–/–*^ hosts and grafted them with BALB/c skin grafts. As shown in Supplementary Fig. 1A, B, while the WT TEa cells accumulated in substantial numbers in the host spleen, LNs, and the skin grafts, the R111A TEa cells failed to survive and enrich at those sites. Furthermore, in an in vitro Treg-mediated suppression assay, in which proliferation of the CD4^+^ Tconv cells to anti-CD3 plus APC stimulation was suppressed by the WT Tregs, the R111A Tregs completely failed to do so, suggesting a profound functional defect of R111A Tregs in vitro (Fig. 1G, H).

### Conditional expression of the R111A mutant selectively in Foxp3^+^ Tregs results in lethal autoimmunity

**Fig. 2:**
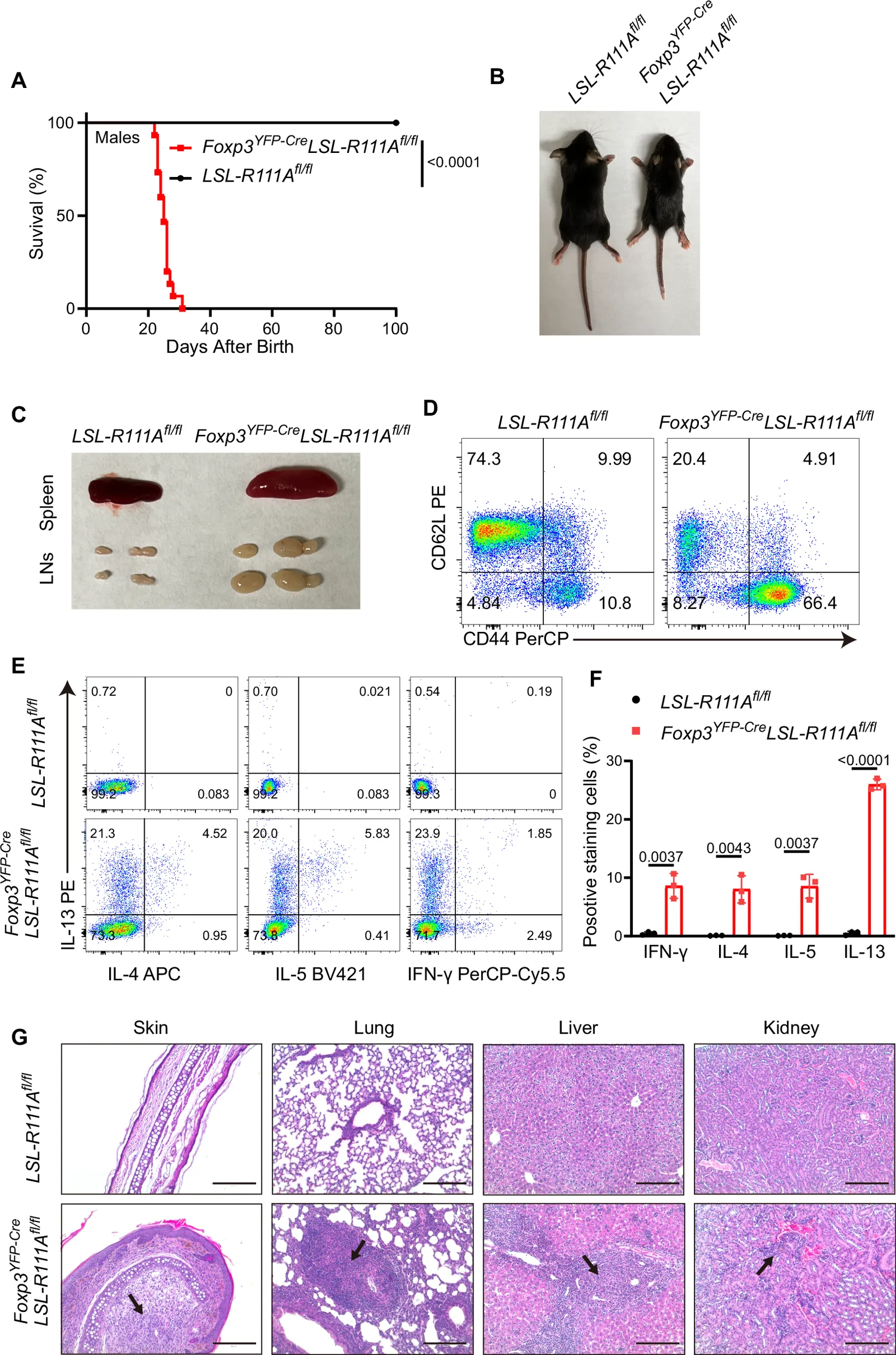
Conditional expression of the R111A mutant in Foxp3^+^ Tregs induces fatal systemic autoimmune diseases. **A** The Kaplan-Meier curve showing overall survival of male *LSL-R111A*^*fl/fl*^ controls and *Foxp3*^*YFP-Cre*^*LSL-R111A*^*fl/fl*^ mice (*n* = 15 mice per group). **B** A representative picture comparing 3-week-old male *LSL-R111A*^*fl/fl*^ and *Foxp3*^*YFP-Cre*^*LSL-R111A*^*fl/fl*^ mice. The *Foxp3*^*YFP-Cre*^*LSL-R111A*^*fl/fl*^ mice were small and failed to thrive as compared to the controls. **C** A representative picture showing enlarged spleen and lymph nodes (LNs) from 3-week-old male *Foxp3*^*YFP-Cre*^*LSL-R111A*^*fl/fl*^ mice as compared to age-matched control *LSL-R111A*^*fl/fl*^ mice. **D** Representative flow cytometry plots showing an effector/memory phenotype of CD4^+^ T cells in spleen of male *Foxp3*^*YFP-Cre*^*LSL-R111A*^*fl/fl*^ mice as opposed to those in control male *LSL-R111A*^*fl/fl*^ mice. Representative flow cytometry plots (**E**) and relative percentages (**F**) of cytokine-producing CD4^+^ T cells in the spleen of male *Foxp3*^*YFP-Cre*^*LSL-R111A*^*fl/fl*^ *and LSL-R111A*^*fl/fl*^ mice (*n* = 3 mice per group). **G** Histopathology of indicated tissues and organs from age-matched control male *LSL-R111A*^*fl/fl*^ *and Foxp3*^*YFP-Cre*^*LSL-R111A*^*fl/fl*^ mice. Hematoxylin & Eosin staining (H&E), scale bar = 200 µm; ×200 magnification. Arrows indicate areas of extensive immune cell infiltration and tissue damage. Data are presented as mean ± SD. Male mice at 3-4 weeks old were used for all analyses unless otherwise indicated. Results are representative of at least 3 independent experiments (**D**–**F**) or a summary of 3 independent experiments (**A**). The *P* values are determined by the log-rank test (**A**) or two-tailed unpaired Student’s *t* test (**F**).

To study how Regnase-1 functionally regulates Foxp3^+^ Tregs in vivo, we bred the *LSL-R111A*^*fl/fl*^ mice with *Foxp3*^*YFP-Cre*^ mice and generated the *Foxp3*^*YFP-Cre*^*LSL-R111A*^*fl/fl*^ mice, in which the R111A mutant was selectively expressed in Foxp3^+^ Tregs. Surprisingly, all male *Foxp3*^*YFP-Cre*^*LSL-R111A*^*fl/fl*^ mice uniformly died of autoimmune diseases by ~4 weeks of age (Fig. 2A), a striking phenotype that resembles that of the Scurfy mice [21]. As shown in Fig. 2B, the male *Foxp3*^*YFP-Cre*^*LSL-R111A*^*fl/fl*^ mice failed to thrive, with markedly enlarged spleen and lymph nodes (LNs) (Fig. 2C). Phenotypically, an overwhelming majority of splenic CD4^+^ T cells was CD44^hi^CD62L^–^ ( ~ 70%), a typical effector/memory cell phenotype, which contrasted sharply to those from control mice (male *LSL-R111A*^*fl/fl*^ mice) (Fig. 2D). Analysis of the CD8^+^ T cells in male *Foxp3*^*YFP-Cre*^*LSL-R111A*^*fl/fl*^ mice revealed a similar effector/memory phenotype (Supplementary Fig. 1C). Additionally, CD4^+^ T cells from the male *Foxp3*^*YFP-Cre*^*LSL-R111A*^*fl/fl*^ mice expressed increased levels of proinflammatory cytokines, including IFN-γ, IL-4, IL-5, and IL-13 (Fig. 2E, F). Similarly, the CD8^+^ T cells in the male *Foxp3*^*YFP-Cre*^*LSL-R111A*^*fl/fl*^ mice also expressed elevated levels of perforin, granzyme A, granzyme B, and IFN-γ (Supplementary Fig. 1D, E). Histologically, the male *Foxp3*^*YFP-Cre*^*LSL-R111A*^*fl/fl*^ mice developed multi-organ inflammation, involving the skin, lung, liver and the kidney, where intense immune cell infiltration and extensive tissue damage were observed (Fig. 2G), suggesting the presence of systemic autoimmune diseases.

### Foxp3^+^ tregs expressing the R111A mutant exhibit survival defects and are depleted in the periphery

**Fig. 3:**
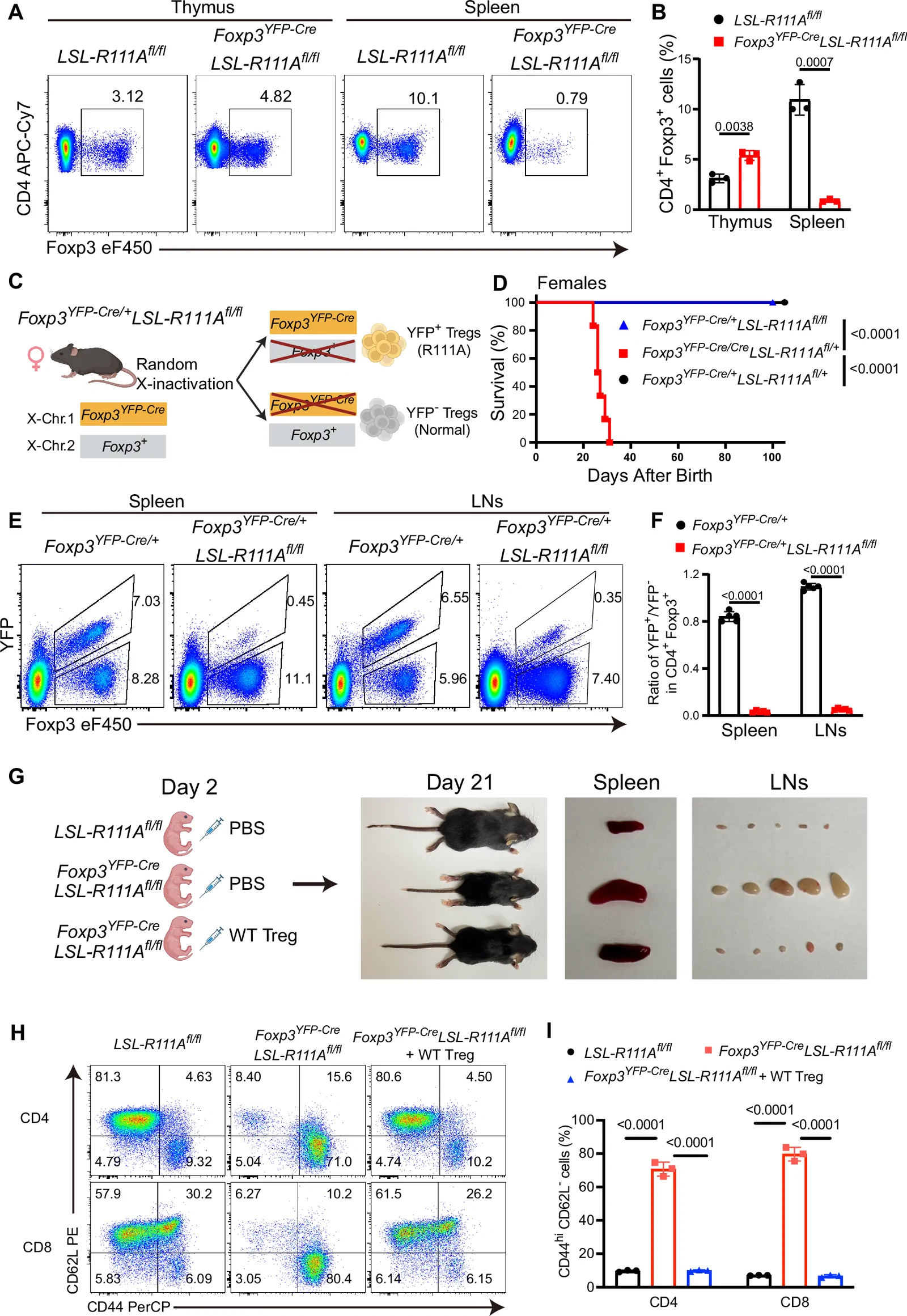
Foxp3^+^Tregs show intrinsic defects in mice expressing the R111A mutant and are depleted in the spleen and LNs. **A** Flow cytometry plot showing Foxp3^+^CD4^+^ Tregs in the thymus versus spleen of 3-week-old male *LSL-R111A*^*fl/fl*^ and *Foxp3*^*YFP-Cre*^*LSL-R111A*^*fl/fl*^ mice. **B** Bar graphs showing the relative percentage of Foxp3^+^ Tregs in CD4^+^ T cells in thymus and spleen (*n* = 3 mice). **C** A cartoon rendering showing mosaic Treg populations in female *Foxp3*^*YFP-Cre/+*^*LSL-R111A*^*fl/fl*^ mice, which express *YFP-Cre* and R111A in half of the Treg cells due to the X-linked *Foxp3*^*YFP-Cre*^ allele and random X-inactivation. **D** Kaplan-Meier curve of female *Foxp3*^*YFP-Cre/+*^*LSL-R111A*^*fl/fl*^, *Foxp3*^*YFP-Cre/+*^*LSL-R111A*^*fl/+*^, and *Foxp3*^*YFP-Cre/YFP-Cre*^*LSL-R111A*^*fl/+*^ mice (*n* = 6 mice per group). Female *Foxp3*^*YFP-Cre/YFP-Cre*^*LSL-R111A*^*fl/+*^ homozygous mice with all Tregs expressing the R111A mutant also died at ~4 weeks of age. Flow cytometry plots (**E**) and the relative ratios (**F**) of YFP^+^ and YFP^-^ Treg cells in the spleen and LNs from female *Foxp3*^*YFP-Cre/+*^ and *Foxp3*^*YFP-Cre/+*^*LSL-R111A*^*fl/fl*^ mice (*n* = 5 mice). **G** A schematic diagram showing adoptive transfer of WT Treg cells (left), where FACS-sorted WT Foxp3^+^CD4^+^ Tregs were transferred into 2-day-old hosts (1 × 10^6^ Tregs per mouse), and representative pictures of host mice showing their spleen and LNs at 19 days after Treg transfer (right). Representative flow cytometry plots (**H**) and relative percentages (**I**) of CD44^hi^CD62L^–^ cells in the splenic CD4^+^ and CD8^+^ T cells from male *Foxp3*^*YFP-Cre*^*LSL-R111A*^*fl/fl*^ mice with or without transferring WT Treg cells (*n* = 3 mice). Data in the bar graphs are presented as mean ± SD. Results are representative of at least 3 independent experiments (**A**, **B**, **G**–**I**) or pooled from 2 independent experiments (**D**, **E**, **F**). Statistical significance was determined by two-tailed unpaired Student’s *t* test (**B**, **F**), log-rank test (**D**), or one-way ANOVA with Tukey’s multiple comparison test (**I**).

To determine the underlying mechanisms of the lethal autoimmune phenotype, we initially focused on the Foxp3^+^ Tregs and examined whether they were defective. As shown in Fig. 3A, B and Supplementary Fig. 2A, Foxp3^+^ Tregs in the thymus were comparable between control (male *LSL-R111A*^*fl/fl*^) and the male *Foxp3*^*YFP-Cre*^*LSL-R111A*^*fl/fl*^ mice from 7 days to 3 weeks of age, suggesting that the development of Tregs was not altered after transgenic expression of the R111A mutant. Strikingly, Tregs in the spleen of male *Foxp3*^*YFP-Cre*^*LSL-R111A*^*fl/fl*^ mice were largely depleted as compared to the controls. Similar findings were also found in the peripheral LNs (data not shown). Moreover, in female *Foxp3*^*YFP-Cre/+*^*LSL-R111A*^*fl/+*^ mice (with two *Foxp3* alleles), in which expression of the R111A mutant (marked by YFP) depends on random X-inactivation (Fig. 3C), we observed that the *Foxp3*^*YFP-Cre*^ homozygous females (*Foxp3*^*YFP-Cre/YFP-Cre*^*LSL-R111A*^*fl/+*^) died of systemic autoimmunity in a similar kinetics as male *Foxp3*^*YFP-Cre*^*LSL-R111A*^*fl/fl*^ mice (by ~4 weeks of age), while all the *Foxp3*^*YFP-Cre*^ hetero females (*Foxp3*^*YFP-Cre/+*^*LSL-R111A*^*fl/+*^ or *Foxp3*^*YFP-Cre/+*^*LSL-R111A*^*fl/fl*^) survived long-term without any signs of autoimmune diseases (Fig. 3D). As expected, flow cytometry analysis of Foxp3^+^ Tregs in the spleen and LNs of female *Foxp3*^*YFP-Cre/+*^ mice identified 2 subsets of Foxp3^+^ Tregs (i.e., YFP^+^ and YFP^–^), approximately in a 1 to 1 ratio (Fig. 3E, F). Remarkably, the YFP^+^ subset in *Foxp3*^*YFP-Cre/+*^*LSL-R111A*^*fl/fl*^ mice (expressing the R111A mutant) was selectively depleted, while the YFP^–^ subset (lack of R111A expression) remained intact (Fig. 3E, F). Of the remaining YFP^+^ Tregs in the female *Foxp3*^*YFP-Cre/+*^*LSL-R111A*^*fl/fl*^ mice ( < 0.5%), expression of Treg-associated markers (CTLA-4, Helios, NRP-1, PD-1, CD73) was markedly reduced as compared to the YFP^-^ control Tregs (Supplementary Fig. 2B); they also failed to acquire the CD44^hi^ effector Treg phenotype (Supplementary Fig. 2C) and completely lost their proliferative capacity when assessed by the Ki67 staining (Supplementary Fig. 2D, E).

We further generated bone marrow chimeric mice, where bone marrow cells from WT B6 mice and male *Foxp3*^*YFP-Cre*^*LSL-R111A*^*fl/fl*^ mice were mixed at a 1:1 ratio (marked by congenic CD45 markers) and injected into lethally irradiated syngeneic B6 hosts (10 million cells per host). Eight weeks later, Foxp3^+^ Tregs in the CD4^+^ cell fraction were examined by flow cytometry (Supplementary Fig. 2F). As shown in Supplementary Fig. 2G, H, Foxp3^+^ T cells were readily identified in the spleen of host B6 mice reconstituted with WT bone marrow cells, but were absent in those constituted with the *Foxp3*^*YFP-Cre*^*LSL-R111A*^*fl/fl*^ bone marrow cells, suggesting that the defect in *Foxp3*^*YFP-Cre*^*LSL-R111A*^*fl/fl*^ mice is Treg-intrinsic in the periphery. To further probe this notion, we FACS-sorted WT Tregs from Foxp3-GFP reporter mice, adoptively transferred them into 2-day-old male *Foxp3*^*YFP-Cre*^*LSL-R111A*^*fl/fl*^ mice (1 million cells/mouse), and monitored them for signs of autoimmunity 19 days later. The male *LSL-R111A*^*fl/fl*^ and *Foxp3*^*YFP-Cre*^*LSL-R111A*^*fl/fl*^ mice without Treg transfer were included as controls for comparison. As expected, the male *Foxp3*^*YFP-Cre*^*LSL-R111A*^*fl/fl*^ mice without Treg transfer developed prominent lymphadenopathy (enlarged spleen and LNs) (Fig. 3G), and most T cells (both CD4^+^ and CD8^+^) in the spleen acquired the effector/memory phenotype (Fig. 3H, I), suggesting the presence of ongoing autoimmune diseases. In stark contrast, in male *Foxp3*^*YFP-Cre*^*LSL-R111A*^*fl/fl*^ mice transferred with WT Tregs, those signs of autoimmune changes were strongly inhibited, showing a phenotype that was similar to the male *LSL-R111A*^*fl/fl*^ control mice (Fig. 3G, H, I).

### HyperTRIBE screening for Regnase-1-targeted mRNAs in the life and death of T cells

**Fig. 4:**
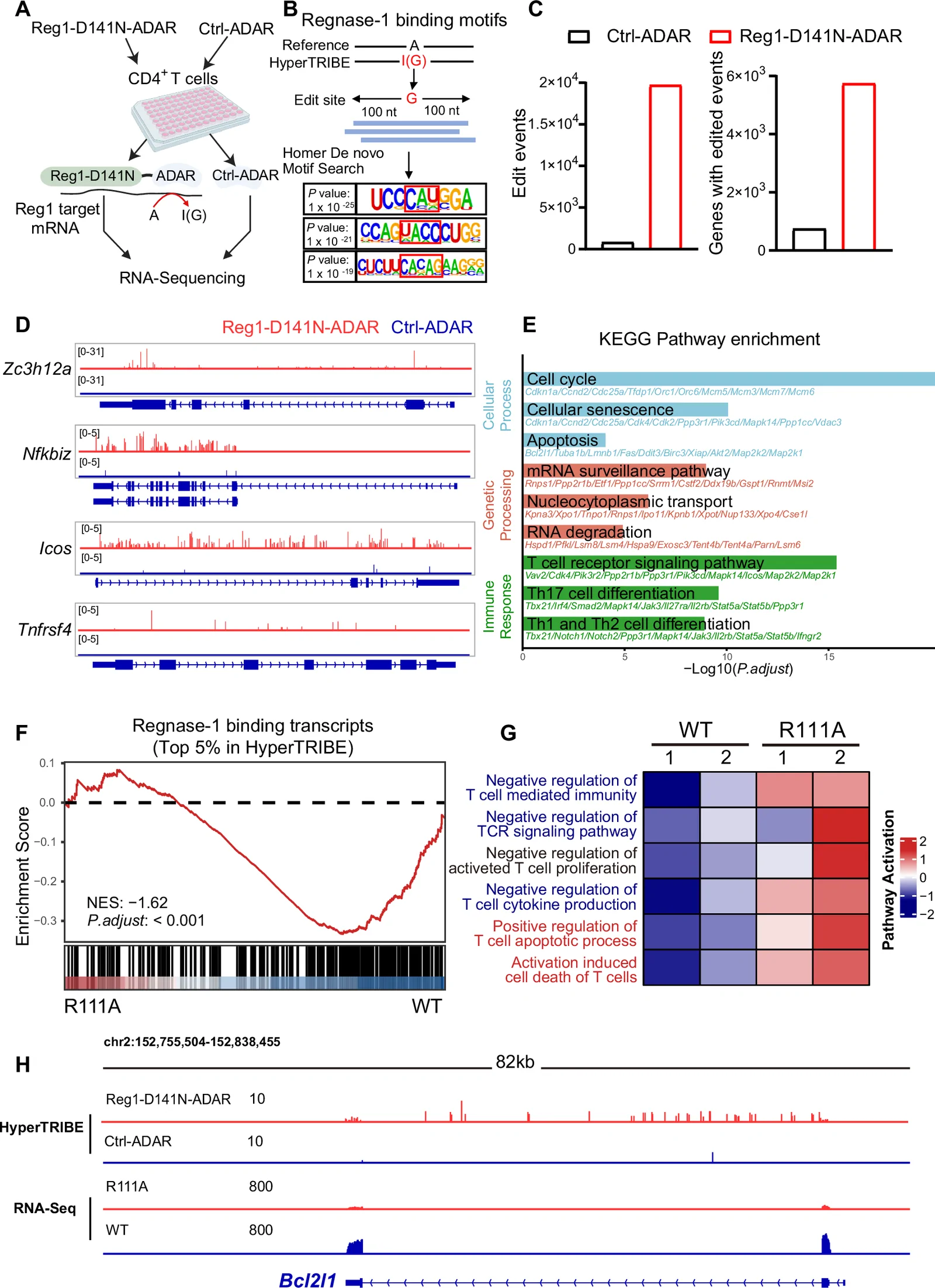
HyperTRIBE careening of mRNA species that are targeted by the R111A mutant. **A** Experimental design of the HyperTRIBE assay, where putative mRNAs that were bound by the nuclease-dead Regnase-1 mutant (Reg1-D141N) were identified by ADAR-mediated conversion of adenosines (A) to inosines (I), and then read by high throughput RNA-seq. ADAR alone group was included as controls to identify specific ADAR-edited events in activated CD4^+^ T cells. **B** A schematic illustration of HOMER de novo motif analysis, extending 100 nt on both sides at edited sites in selected windows and showing enrichment of consensus sequences that match Regnase-1 binding motif as references. **C** The number of edited sites and their corresponding genes identified by the HyperTRIBE assay in activated CD4^+^ T cells, showing that some genes are edited at multiple sites. **D** Alignments of genes *Zc3h12a, Nfkbiz, Icos, Tnfrsf4*, which are known targets of Regnase-1, showing extensive edits in the HyperTRIBE assay, demonstrating the validity of the assay. **E** KEGG pathway enrichment analysis of mRNA species that were targeted by Regnase-1 in the HyperTRIBE assay, showing biological processes that involve cell cycle, cell survival, activation, and differentiation. **F** GSEA analysis showing that the top mRNA targets identified by the HyperTRIBE assay (285 genes with top 5% edit frequency) were enriched among genes that were markedly downregulated in R111A CD4^+^ T cells. **G** A heatmap plot showing marked differences in gene set enrichment scores between WT and R111A CD4^+^ T cells identified by Gene Set Variation Analysis (GSVA). Biological processes of the Gene Ontology (GO) database were used for pathway analysis, showing negative regulation pathways involved in T cell activities and positive regulation of T cell death in R111A CD4^+^ T cells. **H** Gene tracks showing the *Bcl2l1* edited events identified by the HyperTRIBE assay. The alignment of RNA-seq reads of WT or R111A CD4^+^ T cells was also shown. Reg1-D141N–ADAR showed ample edited events in the *Bcl2l1* gene as compared to the Ctrl-ADAR, and *Bcl2l1* gene expression was markedly decreased in R111A CD4^+^ T cells identified by RNA-seq.

To screen for the Regnase-1-targeted mRNA species, we performed a series of HyperTRIBE assays (Hyper efficient Targets of RNA-binding proteins Identified by enzymatic RNA Editing), in which the nuclease-dead Regnase-1 mutant (Reg1-D141N), which binds but fails to degrade mRNA [22]. was genetically linked to the catalytic domain of the highly efficient Adenosine Deaminase Acting on RNA (ADAR) [23]. Thus, in Reg1-D141N-bound mRNA species, the ADAR will convert adjacent adenosine to inosine, which can be identified and mapped through high-throughput RNA-sequencing (Fig. 4A, B) [23]. As the R111A-expressing Tregs have obvious survival defects, we therefore used WT CD4^+^ T cells as surrogates and transduced the Reg1-D141N-ADAR construct into activated CD4^+^ T cells using a retroviral vector [24]. We then performed de novo motif search (HOMER) on the 200 bp sequences flanking the known edited sites and showed that the enriched consensus motifs (Fig. 4B) are highly consistent with the reported Regnase-1 binding motifs, thus further validating the specificity of our HyperTRIBE assay.

As shown in Fig. 4C, we observed ~20,000 edited events in CD4^+^ T cells as compared to the ADAR-Ctrl group (no RNA binding), which corresponded to ~6000 edited genes, suggesting that some genes may encompass multiple edited sites. Indeed, some of the known Regnase-1 target genes, including *Zc3h12a*, *Nfkbiz*, *Icos*, and *Tnfrsf4*, showed ample edited sites, as shown in the HyperTRIBE assay (Fig. 4D). Among the mRNA species that were edited in the HyperTRIBE assay, they could be classified into 3 broad groups, as revealed by the KEGG pathway enrichment analysis, i.e., cellular process, genetic processing, and immune responses, in which apoptosis, RNA degradation, and TCR signaling were prominently featured in each category (Fig. 4E). We also performed bulk RNA-seq on WT and R111A-expressing CD4^+^ T cells, and by integrating the HyperTRIBE-edited transcripts with the transcriptional profiling, we focused on the top 5% of the most edited transcripts and examined their expression dynamics in the RNA-seq dataset. As shown in Fig. 4F, Gene Set Enrichment Analysis (GSEA) revealed that the signature Regnase-1 targets in WT controls were significantly downregulated in R111A-expressing T cells. Furthermore, the targeted genes with high expression levels in R111A-expressing T cells were related to suppression of T cell activation and cell death pathways (Fig. 4G). Thus, expression of the R111A mutant has striking effects on mRNA species related to T cell survival and T cell fates.

Assessments of gene expression levels between WT CD4^+^ T cells and R111A-expressing cells, as depicted in the volcano plot (Supplementary Fig. 3A), showed that the significantly downregulated genes in R111A CD4^+^ T cells were mostly associated with DNA replication and anti-apoptotic pathways, while upregulated genes were largely involved in cell cycle arrest and pro-apoptotic pathways (Fig. 4G). KEGG pathway enrichment analysis revealed that the differentially expressed genes were mostly related to TCR signaling, apoptosis, and cell senescence (Supplementary Fig. 3B). Interestingly, by integrating our RNA-seq dataset, the public dataset on WT and conditional *Regnase-1* KO T cells [11, 25]. and the known T cell apoptosis pathways in a Venn diagram, *Bcl2l1* was identified as the most impacted gene (Supplementary Fig. 3C). We then examined the *Bcl2l1* gene transcripts from the HyperTRIBE assay and found that the *Bcl2l1* transcripts indeed showed ample edited sites by Reg1-D141N-ADAR (Fig. 4H). Alignment with the RNA-seq data revealed that the *Bcl2l1* mRNA was highly expressed in WT CD4^+^ T cells (2 exons shown), but not in R111A-expressing CD4^+^ T cells, and the edited sites were present at both introns and exons (Fig. 4H). Collectively, these data suggest that the *Bcl2l1* mRNA is likely a direct target of R111A, where R111A may prevent Bcl-xL protein expression and cell survival.

### Regnase-1 degrades *Bcl2l1* mRNA via its stem loop structure and blocks Bcl-xL expression in T cells

**Fig. 5:**
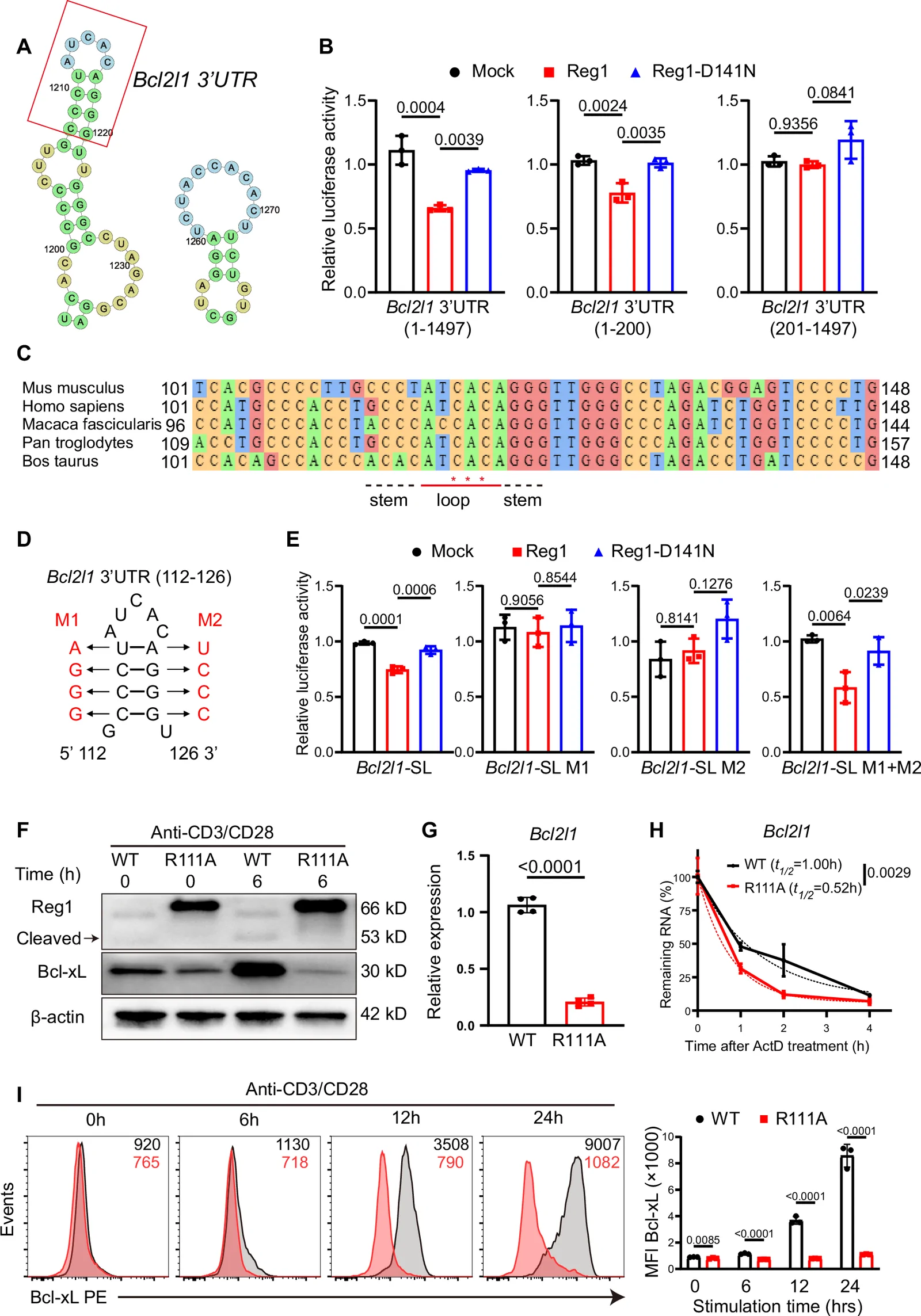
Regnase-1 degrades *Bcl2l1* mRNA by recognizing its stem-loop structure and inhibits Bcl-xL expression in activated T cells. **A** RNAfold prediction of *Bcl2l1* mRNA secondary structures using the minimum free energy mode, showing a stem-loop structure at its 3’UTR region. **B** Luciferase reporter assay in which the HEK293 cells were transfected with pmirGLO reporter plasmids containing various lengths of the *Bcl2l1* 3’UTR, together with expression plasmids for wild-type Regnase-1 (Reg1), nuclease-dead Reg1-D141N mutant, or empty control (Mock) (*n* = 3 independent replicates). **C** A schematic diagram showing Regnase-1 recognition sequences at the stem-loop structure of mouse *Bcl2l1*, together with sequence alignment of four other species. Stem-loop structure at the 3’UTR was predicted using the RNAfold software. Red asterisks indicate Regnase-1 recognition sites at the loop sequences. **D** A schematic diagram showing the mouse *Bcl2l1* stem-loop structure recognized by Regnase-1. M1 and M2 depict mutation sites that abrogate stem-loop structures. **E** Luciferase reporter assay was performed using pmirGLO plasmids containing either full length stem loop (SL) or mutated stem-loop sequences (M1, M2, or M1 + M2) as shown in (**D**), where HEK293 cells were transfected with pmirGLO reporter plasmids together with expression plasmids including empty control (Mock), wild-type Regnase-1 (Reg1) or nuclease-dead Reg1-D141N mutant (*n* = 3 independent replicates). **F** Immunoblot analysis comparing expression of Bcl-xL, Regnase-1 and its cleaved fragment in FACS-sorted WT and R111A CD4^+^ T cells with or without anti-CD3 plus anti-CD28 stimulation for 6 h. β-actin was used as a loading control. **G** RT-qPCR analysis of *Bcl2l1* gene expression in splenic WT and R111A CD4^+^ T cells 6 h after anti-CD3/anti-CD28 stimulation (*n* = 4 independent replicates). **H** *Bcl2l1* mRNA stability assay in FACS-sorted splenic CD4^+^ WT and R111A T cells. Cells were stimulated with anti-CD3/anti-CD28 for 6 h, followed by the addition of actinomycin D (ActD). The remaining mRNA relative to the 0 h time point was measured by RT-qPCR. The half-life was calculated using a nonlinear regression curve fit with one phase decay and shown (*n* = 3 independent replicates per time point). **I** Flow cytometry analysis (left) showing Bcl-xL expression in WT and R111A CD4^+^ T cells at different time points after anti-CD3/anti-CD28 stimulation, the bar graphs (right) summarize Bcl-xL expression in WT and R111A CD4^+^ T cells at indicated time points (*n* = 3 independent replicates). Data are presented as mean ± SD. Results are representative of at least three independent experiments (**B**, **E**, **F**, **H**, **I**) or pooled from 2 independent experiments (**G**). Statistical significance was determined using two-tailed unpaired Student’s *t* test (**G**, **I**), one-way ANOVA with Tukey’s multiple comparison test (**B**, **E**) or two-way ANOVA (**H**).

Regnase-1 is known to degrade mRNA species that possess a stem loop structure at the 3’UTR region [9]. We therefore analyzed the *Bcl2l1* mRNA sequence using the RNAfold platform and found putative stem loop motifs consisting of purine (A or G) and pyrimidine (C or U) bases followed by multiple GC pairs, occupying approximately 1200 nucleotides (nt) at the 3’ UTR region (Fig. 5A and Supplementary Fig. 4A). We then constructed a luciferase reporter assay where different fragments of the *Bcl2l1* 3’UTR were cloned into the reporter vector (Supplementary Fig. 4B). These reporters were co-transfected into HEK293 cells along with expression vectors encoding wild-type Regnase-1, the R111A mutant, the nuclease-dead D141N mutant, or an empty control (Mock). In this setting, if the *Bcl2l1* reporter vector contained a stem loop, the luciferase reporter activities would be inversely correlated with the nuclease activity of Regnase-1 (Supplementary Fig. 4C, D) [10]. As shown in Fig. 5B, a substantial reduction of luciferase activities was observed in HEK293 cells carrying the wild-type Regnase-1, along with the full-length *Bcl2l1* 3’UTR fragment (1497 nt), or the 1-200 nt fragment, but not the 201-1497 fragment, while the nuclease-dead D141N mutant failed to show any reduced luciferase activities under any conditions, suggesting that the stem loop structure that the Regnase-1 binds is likely located at the 1-200 nt region.

Alignment of the *Bcl2l1* 3’UTR sequences from different species revealed a highly conserved stem loop structure (Fig. 5C), which was depicted in Fig. 5D and in Supplementary Fig. 4A. To examine the roles of this stem motif, we introduced a series of point mutations to disrupt the stem-loop structure and tested the susceptibility of those mutants to Regnase-1 in the same luciferase reporter assay (Fig. 5D). As shown in Fig. 5E, while the wild-type Regnase-1 substantially reduced the luciferase activities of the reporter possessing an intact stem loop, this reduction was abolished when base-pairing was disrupted by mutations of either side of the stem loop (M1 or M2). Notably, restoring the stem-loop structure through compensatory mutations (M1 + M2) largely restored the susceptibility to Regnase-1-mediated degradation (Fig. 5E). Clearly, these data demonstrate that Regnase-1 can directly bind and degrade the *Bcl2l1* mRNA via the conserved stem-loop structure at the 3’UTR region. To further ascertain this notion, we activated WT B6 and R111A-expressing CD4^+^ T cells in vitro with anti-CD3/CD28 and assessed the concurrent expression of both Regnase-1 and Bcl-xL in the activated CD4^+^ T cells. As shown in Fig. 5F, as early as 6 h after T cell activation, Regnase-1 was almost completely degraded in WT CD4^+^ T cells, while the R111A mutant remained expressed at high levels. Interestingly, Bcl-xL was highly induced in WT CD4^+^ T cells but strongly reduced in R111A expressing CD4^+^ T cells, showing a striking inverse relationship between Regnase-1 and Bcl-xL expression (Fig. 5F). The levels of *Bcl2l1* mRNA were also significantly downregulated in activated R111A CD4^+^ T cells (Fig. 5G). Furthermore, analysis of *Bcl2l1* mRNA stability showed that the half-life of *Bcl2l1* mRNA was also markedly shortened in R111A-expressing CD4^+^ T cells (Fig. 5H). Additionally, flow cytometry analysis revealed strong Bcl-xL upregulation in WT CD4^+^ T cells following anti-CD3/CD28 stimulation, which was not observed in activated R111A CD4^+^ T cells (Fig. 5I). Collectively, those data demonstrate that the R111A mutant directly degrades *Bcl2l1* mRNA, preventing Bcl-xL expression in activated T cells.

### Foxp3^+^ tregs expressing the R111A mutant fail to upregulate Bcl-xL in vitro and fail to respond to IL-2 in vivo

**Fig. 6:**
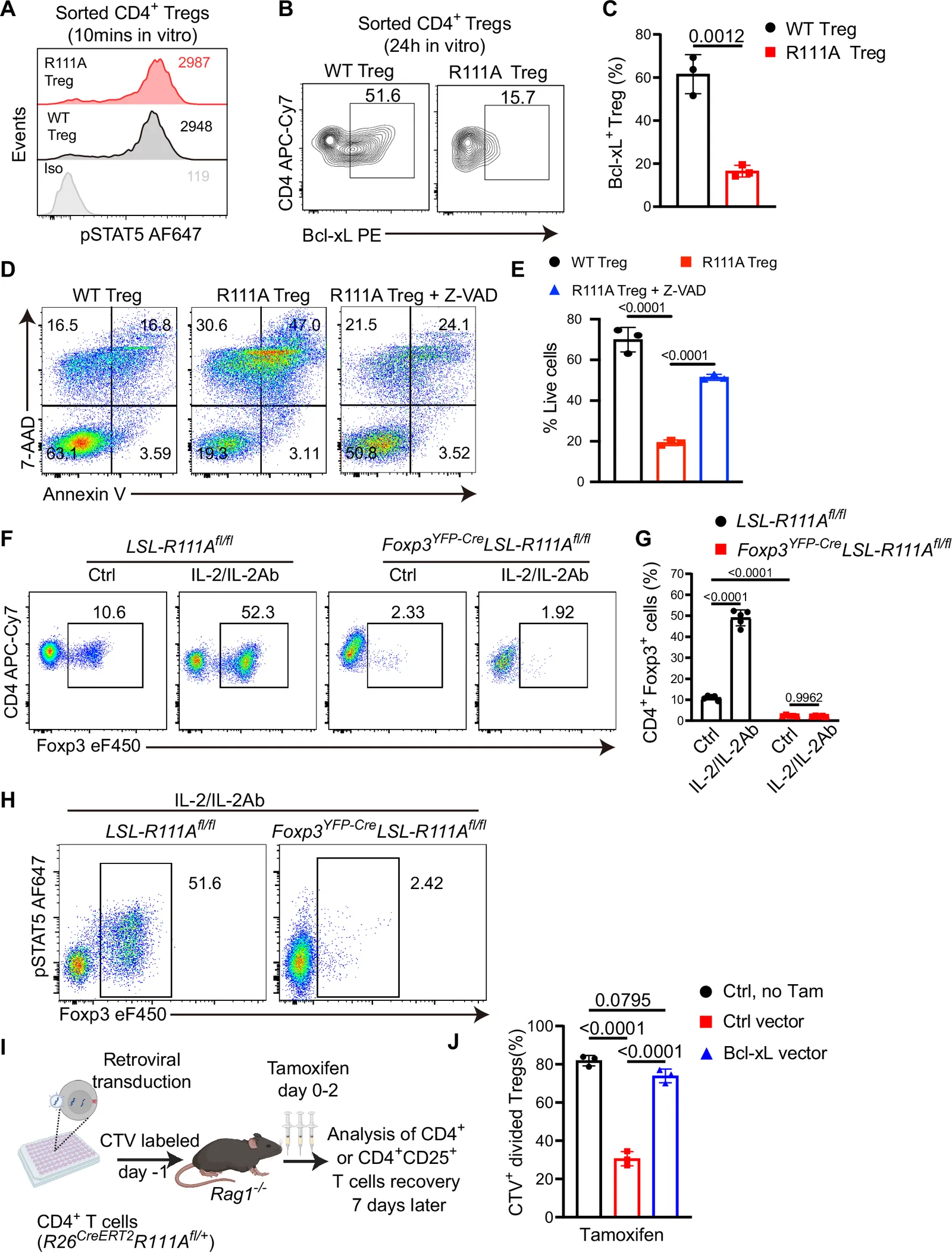
Foxp3^+^Tregs expressing the R111A mutant fail to express Bcl-xL and die of apoptosis. **A** Flow cytometry plots showing phosphorylated STAT5 (pSTAT5) in sorted GFP^+^CD4^+^CD25^+^ Tregs from Foxp3-GFP reporter mice (WT Treg) and tamoxifen-treated *Cd4*^*CreERT2*^*LSL-R111A*^*fl/fl*^ mice (R111A Treg). The sorted Tregs were briefly stimulated with anti-CD3/anti-CD28 and IL-2 and stained for pSTAT5. Treg cells stained with the isotype control Ab were used as a control. Representative flow cytometry plots (**B**) and relative percentages (**C**) of Bcl-xL^+^ Tregs from Foxp3-GFP reporter mice (WT Treg) and tamoxifen-treated *Cd4*^*CreERT2*^*LSL-R111A*^*fl/fl*^ mice (R111A Treg) following stimulation with anti-CD3/anti-CD28 and IL-2 for 24 h (*n* = 3 independent replicates). Flow cytometry plots (**D**) and bar graphs (**E**) showing the Annexin V and 7-AAD staining and percentages of live cells of sorted CD4^+^CD25^+^GFP^+^ Tregs from Foxp3-GFP reporter mice (WT Treg) and tamoxifen-treated *Cd4*^*CreERT2*^*LSL-R111A*^*fl/fl*^ mice (R111A Treg) cultured with or without the pan-caspase inhibitor Z-VAD-FMK (Z-VAD) (*n* = 3 independent replicates). The 7-day-old control male *LSL-R111A*^*fl/fl*^ *mice* and *Foxp3*^*YFP-Cre*^*LSL-R111A*^*fl/fl*^ mice were treated with an IL-2/IL-2Ab complex for 4 days, and expansion of Foxp3^+^ Tregs in the spleen of treated mice was determined by flow cytometry (**F**), mice without IL-2/IL-2Ab injection were used as baseline controls for comparison. The relative percentages of Tregs in CD4^+^ T cells in the treated and control mice were shown in the bar graphs (**G**). (*n* = 5 mice). **H** Representative flow cytometry plots showing pSTAT5 expression in Foxp3^+^ Tregs from 7-day-old male *LSL-R111A*^*fl/fl*^ and *Foxp3*^*YFP-Cre*^*LSL-R111A*^*fl/fl*^ mice treated with IL-2/IL-2Ab complex. **I** Experimental design of retroviral transduced CD4^+^ T cells expressing the Bcl-xL. CD4^+^ T cells isolated from *R26*^*CreERT2*^*R111A*^*fl/+*^ mice were activated and transfected with Bcl-xL-mCherry or Ctrl-mCherry, labeled with CTV, and then adoptively transferred into *Rag1*^*–/–*^ mice. Some recipient mice were treated with tamoxifen for 3 days to induce R111A expression in the transferred T cells, and the CD4^+^CD25^+^mCherry^+^ T cells in host mice were analyzed using flow cytometry on day 7 after transfer. **J** Bar graphs showing the percentage of CTV^+^ dividing T cells among the CD4^+^CD25^+^mCherry^+^ T cells 7 days after transfer (*n* = 3 mice per condition). Data are presented as mean ± SD. All results are representative of three independent experiments (**A**–**E**, **H**) or pooled from 2-3 independent experiments (**F**, **G**, **I**, **J**). Statistical significance was determined by two-tailed unpaired Student’s *t* test (**C**), one-way ANOVA with Tukey’s multiple comparison test (**E**, **G**) or two-way ANOVA Šidák’s multiple comparison test (**J**).

To determine whether the R111A-expressing Tregs would show a similar defect in Bcl-xL expression, we FACS-sorted Tregs from WT Foxp3-GFP reporter mice and tamoxifen-treated *Cd4*^*CreERT2*^*LSL-R111A*^*fl/fl*^ mice, briefly stimulated them in vitro with anti-CD3/anti-CD28 in the presence of IL-2, and examined the expression of phosphorylated STAT5 (pSTAT5) as well as Bcl-xL at different time points. As shown in Fig. 6A, pSTAT5 expression was comparable between WT Tregs and R111A Tregs following a brief exposure to IL-2 (10 min), suggesting that the proximal IL-2 signaling is not altered in R111A Tregs. Interestingly, while the WT Tregs strongly upregulated Bcl-xL expression 24h later, the R111A Tregs completely failed to do so (Fig. 6B, C). Furthermore, when the FACS sorted Tregs were cultured in vitro in the presence of IL-2, we observed precipitous cell death of R111A Tregs 24 h later ( ~ 70%), as shown by Annexin V and 7-AAD staining, which could be rescued, to a large extent, by the addition of the pan-caspase inhibitor Z-VAD (Fig. 6D, E), suggesting that the R111A Tregs exhibit survival defects and readily die of apoptosis.

To examine Treg survival in vivo and their responsiveness to IL-2, we treated the 7-day-old male *LSL-R111A*^*fl/fl*^ control mice and male *Foxp3*^*YFP-Cre*^*LSL-R111A*^*fl/fl*^ mice with an IL-2/IL-2Ab complex in vivo, which is known to expand the Treg pool in vivo [26]. and Tregs in the host spleen were determined by flow cytometry 5 days later. As shown in Fig. 6F, G, Tregs expanded for ~5-fold in the control *LSL-R111A*^*fl/fl*^ mice after treatment with the IL-2/IL-2Ab complex. Strikingly, the same IL-2/IL-2Ab complex completely failed to expand Tregs in male *Foxp3*^*YFP-Cre*^*LSL-R111A*^*fl/fl*^ mice. This expansion defect was further corroborated by intracellular staining for pSTAT5 in the Foxp3^+^ fraction, which revealed prominent pSTAT5 induction in control Tregs but absence of pSTAT5 in R111A Tregs (Fig. 6H). Additionally, we overexpressed Bcl-xL in CD4^+^ T cells from *R26*^*CreERT2*^*LSL-R111A* ^*fl/+*^ mice using a retroviral vector (mCherry^+^) and the transduced CD4^+^ T cells were adoptively transferred into the *Rag1*^*–/–*^ hosts and then treated with tamoxifen, followed by flow cytometry analysis on CD4^+^mCherry^+^ T cells (including CD25^+^ and CD25^-^) 7 days later (Fig. 6I). As shown in Fig. 6J, the CD4^+^CD25^+^ fraction of T cells that expressed the Bcl-xL vector divided and survived in vivo, which contrasted to those transduced with the control vector. Altogether, these findings demonstrate that Bcl-xL is a critical target of Regnase-1 in regulating in vivo Treg survival and homeostasis.

## Discussion

Regnase-1, as an RNA-binding protein with ribonuclease activities involved in TCR signaling has been extensively studied [2, 9]. But its exact role in regulating Foxp3^+^ Tregs is less so and remains largely unknown. Studies using the “loss-of-function” mouse models involving either Regnase-1 itself or its upstream regulators (CBM components) give rise to inconclusive and oftentimes confusing results [7, 11, 27]. In the present study, we developed a degradation-resistant R111A mutant model and found that mice conditionally expressing this R111A mutant in all Tregs (male *Foxp3*^*YFP-Cre*^*LSL-R111A*^*fl/fl*^ and female *Foxp3*^*YFP-Cre/YFP-Cre*^*LSL-R111A*^*fl/+*^ mice) died of aggressive autoimmune diseases by 4 weeks of age because of a profound Treg defect in the periphery, thus uncovering a fundamental role for Regnase-1 in regulating Treg cell fate. We provide strong evidence that the defect in mice with R111A-expressing Tregs was Treg-intrinsic, contributing to accelerated decay of *Bcl2l1* mRNA and compromised Treg survival in vivo. This survival defect also rendered the R111A-expressing Tregs unable to expand in response to IL-2 in vivo. Collectively, our data demonstrate a critical role of Regnase-1 in Treg homeostasis and suppressive functions and further suggest that modulating Regnase-1 in Foxp3^+^ Tregs may have important clinical implications in certain immune-mediated diseases where Tregs are prominently involved.

The fatal autoimmune phenotype of *Foxp3*^*YFP-Cre*^*LSL-R111A*^*fl/fl*^ mice is striking and rather unexpected, a phenotype that closely resembles that of the Scurfy mice [21, 28]. which also die of lethal autoimmune diseases at ~4 weeks of age due to defective Tregs. However, the underlying mechanisms for the Treg defects are very different between such two strains. The Treg defect in Scurfy mice is due to genetic mutations at the *Foxp3* gene, leading to the loss of Foxp3, a lineage-specific transcription factor for the genesis of Tregs [29]. thus resulting in a global impairment of Treg development and total absence of Tregs in the thymus as well as in peripheral lymphoid tissues [21]. In the *Foxp3*^*YFP-Cre*^*LSL-R111A*^*fl/fl*^ mice, however, the Treg defect was confined to the peripheral lymphoid tissues, and the intra-thymic Treg development did not seem to be impacted. In fact, Tregs in the thymus of control (male *LSL-R111A*^*fl/f*^) and male *Foxp3*^*YFP-Cre*^*LSL-R111A*^*fl/fl*^ mice were highly comparable, and adoptive transfer of WT Tregs into the male *Foxp3*^*YFP-Cre*^*LSL-R111A*^*fl/fl*^ mice prevented T effector cell activation and also rescued the autoimmune phenotype, findings that clearly support a peripheral Treg defect in those mice. We provided additional data showing that this peripheral Treg defect is the results of the accelerated decay of the *Bcl2l1* mRNA, which subsequently abrogates Bcl-xL expression, a major survival factor for Tregs [30, 31]. as well as their complete loss of IL-2 responsiveness. Apparently, in R111A-expressing Tregs, the proximal IL-2 signaling seems not altered, as revealed by pSTAT5 expression, but IL-2 fails to upregulate Bcl-xL expression. IL-2 is indispensable in Treg survival, expansion, and homeostasis [32]. The loss of IL-2 responsiveness compromises Treg cell survival, leading to the eventual Treg depletion in the periphery. Clearly, this mechanism is as potent as Foxp3 mutations in inducing lethal autoimmune diseases.

The HyperTRIBE assay proves to be instrumental in assessing mRNAs that are selectively targeted by Regnase-1, and in combination with other bioinformatic tools, we were able to provide novel structural insights into the 3’UTR of *Bcl2l1* mRNA [33]. approaches that are also applicable to assessing other mRNA species as well. Although we can’t rule out the involvement of additional mRNAs that are targeted by the Regnase-1 in our model, the destabilization of *Bcl2l1* mRNA clearly plays a critical role in the survival defects of both Tregs and Tconv cells in mice expressing the R111A mutant. This notion is supported by our finding that overexpression of Bcl-xL could largely rescue their survival defects. In multiple other models and experimental conditions, Bcl-xL has been shown to be critical in the survival and expansion of T cells [34]. It has also been shown that transgenic expression of Bcl-xL in T cells substantially prolongs T cell survival, even under sub-optimal activation conditions [35]. data that are consistent with our current findings.

It is well known that Tconv cells and Foxp3^+^ Tregs follow a very different developmental trajectory [18]. Tregs are phenotypically similar to effector/memory cells; they are positively selected and matured in the thymus before being exported to the periphery, a process that relies on productive TCR signaling [36]. We reported before that expression of the R111A mutant in T cells using the *Cd4-Cre* driver disrupts thymic T cell development, with the subsequent development of profound lymphopenia [37]. Hence, our current finding that inducible expression of the same R111A mutant (by tamoxifen administration) spares Tregs in the thymus is highly intriguing. One possible explanation is that Regnase-1 may play a role in thymic Tregs before Foxp3 expression, and therefore, the *Foxp3Cre*-mediated expression of the R111A mutant may exhibit a different mechanism of action. Alternatively, thymic Tregs may possess Regnase-1-independent mechanisms in survival and expansion. Equally interesting is the exquisite sensitivity of peripheral Tregs to Regnase-1 overexpression. Studies in mice carrying the Nur77 reporter, which faithfully reports TCR signaling events, demonstrate constitutive tonic TCR activities in Tregs as opposed to Tconv cells [19]. These basal TCR activities in Tregs may be sufficient to block Regnase-1 to support Treg survival, their IL-2 responsiveness, and Treg homeostasis. This may also explain the lack of notable Treg abnormalities in embryonic Regnase-1 deleted mice [11]. This is a significant area that deserves further investigation in future studies.

Our studies also suggest additional opportunities and challenges in modulating Tregs and Tconv cells for therapeutic purposes. Given the profound effects of the mutant R111A on Tregs, the R111A site is clearly a valid target for therapeutic modulation of Regnase-1, which may provide additional precision than targeting the paracaspase activities of MALT1, which has a multiplicity of substrates [5]. In situations where Treg cell inhibition is desired (e.g., cancer immunotherapies), the challenge is to selectively deliver Regnase-1 cleavage inhibitors to Tregs to inhibit Treg functions but leave T effector cells unaffected. Conversely, in autoimmune diseases and organ transplantation, the challenge is to prevent Regnase-1 from being degraded in T effector cells while preserving Tregs to shift the immune responses toward tolerance. Clearly, further progress in this area demands novel mechanistic insights and new interventional approaches [38]. Moreover, further studies are also needed to understand how Regnase-1 mutants, including the R111A, would affect other immune cell types, especially B cells and myeloid cells.

## Materials and methods

### Animals

Wild type C57BL/6 (CD45.2, #00066), B6.SJL-*Ptprc*^a^ *Pepc*^b^*/BoyJ* (B6.CD45.1, #002014), BALB/cJ (#000651), B6.Cg-Tg(Tcra,Tcrb)3Ayr/J (TEa transgenic, #005655), B6.129S7-*Rag1*^*tm1Mom*^/J (*Rag1*^*–/–*^, #002216), B6(129×1)-Tg(Cd4-cre/ERT2)11Gnri/J (*Cd4*^*CreERT2*^, #022356), B6.129-*Gt(ROSA)26Sor*^*tm1(cre/ERT2)Tyj*^/J (*R26*^*CreERT2*^, #008463), B6.Cg-*Foxp3*^*tm1Kuch*^/J (Foxp3-GFP, #035864), and B6.129(Cg)-*Foxp3*^*tm4(YFP/icre)Ayr*^/J (*Foxp3*^*YFP-Cre*^, #016959) were purchased from the Jackson Laboratory (Bar Harbor, Maine).

The R111A mutant mice, referred here as *LSL-R111A*^*fl/fl*^ on the C57BL/6 background, were generated in our laboratory. Briefly, a targeting construct, consisting of a synthetic promoter CAG, a STOP cassette flanked by two *loxP* sites, and the coding sequences for the R111A mutant, the self-cleaving P2A, and the enhanced GFP (EGFP), was inserted between exon 1 and exon 2 of the *Rosa26* locus. Thus, the *Cre-*mediated recombination would clip off the STOP cassette, allowing the expression of both the R111A mutant and EGFP driven by the CAG promoter. WT B6 CD45.1^+/–^ mice were obtained by crossbreeding WT B6 mice with B6.CD45.1 mice. *LSL-R111A*^*fl/fl*^ mice were crossed with *Cd4*^*CreERT2*^, *R26*^*CreERT2*^ and *Foxp3*^*YFP-Cre*^ mice to generate *Cd4*^*CreERT2*^*LSL-R111A*^*fl/fl*^, *R26*^*CreERT2*^*LSL-R111A*^*fl/+*^ and *Foxp3*^*YFP-Cre/+*^*LSL-R111A*^*fl/+*^ (including male *Foxp3*^*YFP-Cre*^*LSL-R111A*^*fl/fl*^, female *Foxp3*^*YFP-Cre/YFP-Cre*^*LSL-R111A*^*fl/+*^, *Foxp3*^*YFP-Cre/+*^*LSL-R111A*^*fl/+*^, and *Foxp3*^*YFP-Cre/+*^*LSL-R111A*^*fl/fl*^ mice), respectively. *Cd4*^*CreERT2*^*LSL-R111A*^*fl/fl*^ mice were also crossed with TEa mice to generate *Cd4*^*CreERT2*^*LSL-R111A*^*fl/fl*^ TEa mice. B6.CD45.1 mice were crossed with the TEa mice to generate CD45.1^+^ TEa mice [39]. In all experiments, mice with the correct genotypes were randomly selected for functional studies.

All mice were maintained in a specific-pathogen-free facility at Houston Methodist Research Institute under a 12-h light-dark cycle at temperatures of 68-79 °F and 30–70% humidity. Both male and female mice at 6–12 weeks of age were used for all experiments unless otherwise specified in the Results or figure legends section. Animal use and care were approved by the Animal Care Committee at the Houston Methodist under the protocol number IS00009238 in accordance with the institutional animal care and use guidelines.

### Antibodies, cell preparations, flow cytometry and cell sorting

The following antibodies were purchased from BioLegend (San Diego, CA): Alexa Fluor 700-anti-CD45 (30-F11, 103128, 1:400), Brilliant Violet 605-anti-TCRβ (H57-597, 109241, 1:200), APC/Cyanine7-anti-CD4 (GK1.5,100414,1:300), BV785-anti-CD4 (GK1.5, 100453, 1:200), PE/Cyanine7-anti-CD4 (GK1.5, 100422, 1:200), FITC-anti-CD8a (53-6.7, 100706, 1:200), PE-anti-TCR Vα2 (B20.1, 127808, 1:200), Brilliant Violet 785-anti-CD8a (53-6.7, 100750, 1:200), Alexa Fluor 700-anti-CD45.2 (104, 109822, 1:200), Brilliant Violet 785-anti-CD45.1 (A20, 110743, 1:100), APC-anti-CD45.1 (A20, 110714, 1:200), PerCP-anti-CD44 (IM7, 103035, 1:300), PE-anti-CD62L (MEL-14, 104408, 1:100), PerCP/Cyanine5.5-anti-CD304 (3E12, 145208, 1:100), PE-Annexin V (640947; 1:10), 7-AAD (420404, 1:20), Alexa Fluor 488-anti-Helios (22F6, 137223, 1:50), PE/Cyanine7-anti-CD73 (TH/11.8, 127224, 1:300), PE-anti-CTLA4 (UC10-4B9, 106306, 1:100), APC-anti-PD1 (MH5A, 143710, 1:200), PE-anti-Ki67 (11F6, 151210, 1:200), PE/Cyanine7-anti Ki67 (11F6, 151218, 1:200), PE/Cyanine7-anti-GFP (FM264G, 338014, 1:20), APC-anti-GFP (FM264G, 338010, 1:20), PE-Donkey anti-rabbit IgG (Poly4064, 406421, 1:400), PerCP/Cyanine5.5-anti-IFNγ (XMG1.2, 505822, 1:200), APC-anti-IL-4 (11B11, 504106, 1:200), Brilliant Violet 421-anti-IL-5 (TRFK5, 504311, 1:200), FITC-anti-Granzyme B (QA16A02, 372206, 1:50), APC-anti-Perforin (S16009A, 154304, 1:20). Antibodies eFluor 450 anti-Foxp3 (FJK-16s, 48-5773-82, 1:200), eFluor 450 anti-Granzyme A (GzA-3G8.5, 48-5831-82, 1:200), and PE-anti-IL-13 (eBio13A, 12-7133-82, 1:200) were obtained from Thermo Fisher Scientific. Alexa Fluor 647-Stat5 (pY694) (Clone 47/Stat5, 612599, 1:5) was from BD Bioscience. Bcl-xL (54H6) Rabbit mAb (#2764, 1:200) was from Cell Signaling Technology.

Single cell suspensions from thymus, lymph nodes, spleen, and skin grafts were prepared as previously reported [40]. For cell surface staining, cells were first incubated with the cell viability dye Zombie Aqua (BioLegend, 423102) to exclude dead cells, followed by an anti-FcγRII/III antibody (BioLegend, 156604) to block nonspecific antibody binding. After washing, cells were incubated with fluorochrome-conjugated antibodies for 20 min at 4 °C. For intracellular staining, cells were fixed and permeabilized with the Cytofix/Cytoperm solution (BD Biosciences, 554714), or the Foxp3 transcription factor Staining Kit (Thermo Fisher Scientific, 00-5523-00), or Phosflow Fix Buffer I/Phosflow Perm Buffer III (BD Biosciences, 557870 & 558050) according to the manufacturers’ instructions, followed by staining with specific antibodies. For cytokine staining, cells were restimulated for 4 h with phorbol 12-myristate 13-acetate (PMA, 50 ng/mL) and ionomycin (550 ng/mL; both from Sigma-Aldrich) in the presence of GolgiStop (BD Biosciences, 554724). After stimulation, cells were fixed and permeabilized, followed by staining with specific intracellular antibodies according to the manufacturer’s instructions.

All samples were acquired using an LSRFortessa flow cytometer (BD Biosciences), and data were analyzed with FlowJo v.10 software (Tree Star). For cell sorting, splenic CD4^+^ T cells were prepared using Dynabeads Untouched Mouse CD4 Cells Kit (Thermo Fisher Scientific, 11415D) from host mice. For mice with a GFP reporter, the GFP^+^ cells were first gated and then further sorted into CD4^+^CD25^+^ Tregs and CD4^+^CD25^-^ conventional T (Tconv) cells using the FACSAria cell sorter (BD Biosciences).

### T cell activation, immunoblotting, and apoptosis assay

CD4^+^ T cells were purified using Dynabeads Untouched Mouse CD4 Cells Kit from the spleen of WT B6 or *Cd4*^*CreERT2*^*LSL-R111A*^*fl/fl*^ mice after tamoxifen administration (75 mg/kg, i.p. for 5 days, Sigma-Aldrich, T5648-5G) and stimulated with PMA (50 ng/mL) plus ionomycin (550 ng/mL) for 15 min in an Eppendorf tube at 2 × 10^6^ cells per milliliter. In another set of experiments, FACS-sorted WT and R111A CD4^+^ T cells were stimulated with plate-coated anti-CD3 (5 µg/mL, clone 145-2C11; BioLegend, 100301) and soluble anti-CD28 (1 µg/mL, 37.51; BioLegend 102101) at 1 × 10^5^ cells per well for 6 h. Cells were then collected and processed for immunoblotting. Briefly, protein extracts from cultured cells were boiled, electrophoresed in 4–15% SDS-PAGE (Bio-Rad, #4561084), transferred onto an PVDF membrane (Thermo Fisher Scientific, 88518), and analyzed by immunoblot with the following specific antibodies: anti-Mcpip1 (R&D, MAB7875, 1:500), anti-Bcl-xL (Cell Signaling Technology, 2764, 1:1000), and anti-β-actin (Cell Signaling Technology, 12262, 1:1000). HRP-conjugated antibodies to mouse or rabbit IgG (Cell Signaling Technology, 7076 or 7074, both 1:2000) were used as secondary antibodies. The target proteins were visualized using enhanced chemiluminescence reagents (Thermo Fisher Scientific, 34076) and the ChemiDoc system (Bio-Rad, 12003153).

For analysis of cell death, FACS-sorted WT and R111A CD4^+^ T cells were stimulated with different concentrations of plate-coated anti-CD3 (0–10 µg/mL) and soluble anti-CD28 (1 µg/mL) at 1 × 10^5^ cells per well with or without 50 ng/mL IL-2 (PeproTech, 212-12). CD4^+^ T cells cultured for 1-2 days were subsequently assessed for apoptotic cell death by staining for Annexin V and 7-AAD and further analyzed by flow cytometry.

For in vitro culture of Tregs, FACS-sorted GFP^+^CD4^+^CD25^+^ WT-Treg (from Foxp3-GFP reporter mice) and R111A-Treg cells (from tamoxifen-treated *Cd4*^*CreERT2*^*LSL-R111A*^*fl/fl*^ mice) were seeded in a 96-well plate in the presence of anti-CD3 (5 µg/mL), anti-CD28 (1 µg/mL) and IL-2 (50 ng/mL) and cultured for 10 min to 1 day. Subsequently, cells were harvested and assessed by flow cytometry for pSTAT5 and Bcl-xL expression. In certain cultures, a pan-caspase inhibitor Z-VAD-FMK was added (30 µM Z-VAD, Selleckchem, #187389-52), and Treg cell survival was assessed by staining with Annexin V and 7-AAD, followed by flow cytometry analysis.

### Adoptive cell transfer assays and skin grafting

WT TEa (CD45.1^+^) and R111A splenic TEa (CD45.2^+^) were isolated and FACS-sorted from CD45.1^+^ TEa and tamoxifen-treated *Cd4*^*CreERT2*^*LSL-R111A*^*fl/fl*^ TEa mice. The sorted WT and R111A TEa cells were mixed at a 1:1 ratio (1 million each) and co-transferred into *Rag1*^*–/–*^ mice, followed by grafting with BALB/c skin grafts the next day. Briefly, a tail skin graft was transplanted onto the back of the recipient. Rejection was defined as >90% of necrosis of the donor skin graft [40]. In some experiments, the skin grafts, host spleen, and lymph nodes were harvested for further phenotyping of T cell subsets by flow cytometry.

### Treg suppression assay in vitro and adoptive Treg transfer

Dynabeads-purified CD4^+^ T cells from the indicated mice were FACS sorted into Tregs and Tconv cells. In the Treg suppression assay, Tregs were sorted from tamoxifen-treated Foxp3-GFP reporter mice (GFP^+^CD4^+^CD25^+^) or *CD4*^*CreERT2*^*LSL-R111A*^*fl/fl*^ mice (GFP^+^CD4^+^CD25^+^, both are CD45.2^+^), while Tconv cells were from B6.CD45.1 mice. Tconv cells were labeled with CellTrace Violet (CTV, Thermo Fisher Scientific, C34557) and mixed with various ratios of WT or R111A Tregs in a 96-well cell culture plate in the presence of anti-CD3 (1.5 μg/ml) and an equal number of antigen-presenting cells (APCs). After 72 h, proliferation of the CTV-labeled Tconv cells was assessed by flow cytometry [41].

For adoptive transfer of Tregs in vivo, WT Tregs were sorted from Foxp3-GFP reporter mice (GFP^+^CD4^+^CD25^+^) using a BD Bioscience FACSAria II cell sorter and 1×10^6^ Treg cells were injected i.p. into each 2-day-old male *Foxp3*^*YFP-Cre*^*LSL-R111A*^*fl/fl*^ mice. Male *LSL-R111A*^*fl/fl*^ and *Foxp3*^*YFP-Cre*^*LSL-R111A*^*fl/fl*^ mice given PBS were included as controls. At 19 days post-injection, the host mice were euthanized and analyzed for signs of autoimmunity.

### Histological assessment

Tissues and organs were procured from 3-week-old male *LSL-R111A*^*fl/fl*^ and *Foxp3*^*YFP-Cre*^*LSL-R111A*^*fl/fl*^ mice, fixed in 10% formaldehyde, and then processed at the Human Tissue Acquisition and Pathology Core at Baylor College of Medicine (Houston, Texas). Tissue sections were examined blindly under light microscopy (Invitrogen EVOS XL, Thermo Fisher Scientific). Photomicrographs were taken at ×200 magnification.

### Generation of bone marrow chimeras

Donor bone marrow cells were isolated from 3-week-old male B6.CD45.1 (CD45.1^+^) and *Foxp3*^*YFP-Cre*^*LSL-R111A*^*fl/fl*^ (CD45.2^+^) mice, mixed at a 1:1 ratio (5 million cells each), and then injected i.v. into lethally irradiated WT B6 CD45.1^+/–^ recipients. Eight weeks later, T cells, including Tregs, in the spleen and lymph nodes of the chimeric mice were examined and compared by flow cytometry.

### HyperTRIBE assay, sequencing, and data analysis

The hyper-efficient Targets of RNA-binding proteins Identified by enzymatic RNA Editing (HyperTRIBE) assay was performed as previously reported [23]. Briefly, the catalytic domain of Adenosine Deaminases Acting on RNA (ADAR) containing a hyperactive E488Q mutation was cloned into the pMYs-IRES-GFP vector (Cell Biolabs Inc, RTV-021) to generate the control vector (Ctrl-ADAR) [42]. The nuclease-death Regnase-1 mutant (Reg1-D141N) was genetically linked to the ADAR to generate the Reg1-D141N-ADAR vector. The vector pMYs-IRES-GFP served as an empty control (Ctrl-GFP). Those expression vectors were transduced into activated T cells using a retroviral-mediated gene transfer method as previously reported [43]. The successfully transduced T cells were further FACS sorted based on GFP expression, and at least 1 × 10^6^ sorted CD4^+^ T cells expressing the GFP were used for RNA extraction with the Direct-zol RNA Microprep Kit (Zymo Research, 11358). RNA concentration was quantified using a NanoDrop Spectrophotometer (Thermo Fisher Scientific, ND-2000), and ~1000ng of RNA per sample was used for library preparation and high-throughput sequencing.

RNA libraries were prepared by firstly depleting ribosomal RNA using DNBSEQ rRNA Depletion Kit (MGI Tech, 1000005953), and the libraries were sequenced by BGI Genomics using the DNBSEQ platform. After filtering low-quality reads using BGI Genomics in house protocols, the data were further processed following the recommended pipeline [23] with some modifications. Briefly, Trimmomatic (0.38) was adapted to a paired-end sequencing model for additional inferior read filtering, and then the filtered data were aligned to the mouse genome (mm10) using the STAR aligner (2.7.11b) [44, 45]. PCR duplicates were identified and removed by Picard (2.8.2) if needed. Uniquely mapped alignments in SAM format were loaded into a MySQL table with genomic coordinates. The edited events were identified using the wtRNA-RNA approach, in which the nucleotides at each position in the test RNA library (Reg1-D141N-ADAR, Ctrl-ADAR) were compared with those of the control RNA library (wt RNA: Ctrl-GFP). Only those edited events with a minimum of 20 read coverage and 5% editing were considered for further analysis. The resulting editing events from Reg1-D141N-ADAR and Ctrl-ADAR were compared against each other to determine the editing sites that were unique to Reg1-D141N-ADAR. Finally, a list of transcripts was created by tabulating all transcripts that contain edited sites. Bioinformatic tool Bedtools was used to locate the edited sites, and these edited events were further visualized in the Integrative Genomics Viewer (IGV) [46]. For de novo motif discovery, edit sites found by HyperTRIBE in 3’UTR regions were extracted and extended to include 100 nt from both sides, serving as the target sequence pool. Overlapping extended sequences were merged into a single sequence. Background sequences with length 201 nt were randomly selected from 3’ UTR regions that were non-overlapping with the target sequence pool. Bioinformatic tool HOMER was used to search for enriched motifs of length 9–15 [47]. Pathway enrichment analysis, including KEGG pathway, Gene Ontology (GO) and Gene Set Enrichment Analysis (GSEA) was performed using the ClusterProfiler program in R [48].

### RNA-sequencing (RNA-seq) and data analysis

Total RNA was prepared from FACS-sorted WT and R111A CD4^+^ T cells with or without anti-CD3 plus CD28 (5 µg/mL + 1 µg/mL) activation for 12 h using the Direct-zol RNA Microprep Kit. PolyA^+^ RNA was isolated, and RNA sequencing was performed by BGI Genomics. After filtering sequencing adaptors and low-quality bases, the clean data were aligned to the mouse genome (mm10) using Bowtie2 (2.4.4), and gene expression level was calculated by RSEM (v1.2.28) [49, 50]. Differentially expressed genes (DEGs) were identified by the DESeq2 program in R with a 2-fold change in expression and an adjusted *P* value < 0.05. Gene expression differences were visualized as a volcano plot with indicated genes by the ggplot2 program in R [51]. Differential gene expressions between wild-type and Regnase-1 conditional knockout in CD4^+^ T cells were retrieved from published datasets [11], and genes annotated in the apoptosis pathway in the KEGG database were also retrieved. Then both are compared with the differentially expressed genes between wild-type and R111A CD4^+^ T cells to identify overlapped genes in 3 gene sets with the ggVennDiagram program in R. Pathway analysis, including KEGG pathway analysis, Gene Set Enrichment Analysis (GSEA) and Gene Set Variation Analysis (GSVA), was conducted using clusterProfiler and the GSVA program in R [48, 52].

### Quantitative RT-PCR

Total RNA from the indicated cultured cells was extracted using the Direct-zol RNA Microprep Kit according to the manufacturer’s instructions. cDNA was synthesized using iScript Reverse Transcription Supermix (Bio-Rad, 1708841) and amplified with specific primers and SsoAdvanced Universal SYBR Green Supermix (Bio-Rad, 1725275) in a CFX96 Real-time PCR detection system (Bio-Rad). Data were normalized to the housekeeping gene *Hprt*, and the relative abundance of transcripts was calculated by the 2^–ΔΔCt^ method. The specific primers used in this study were synthesized by Genewiz and listed as follows: *Bcl2l1*, 5’- GACAAGGAGATGCAGGTATTGG-3’ (forward) and 5’- TCCCGTAGAGATCCACAAAAGT-3’ (reverse); *Hprt*, 5’- AGTACAGCCCCAAAATGGTTA-3’ (forward) and 5’- CTTAGGCTTTGTATTTGGCTTT-3’ (reverse).

### RNAfold prediction of 3’UTR structures of mRNAs

The RNAfold platform from ViennaRNA Package 2.0 was used to predict the secondary structures of 3’UTR regions of interest mRNA [53]. In brief, the 3’UTR regions of mRNA were broken down into 100 nt sequences and analyzed with RNAfold using default parameters. Stem-loop elements that agree with Regnase-1-binding structure motif (stem size >2 bp with at least 1 GC pair, loop size = 3–6 nt with a YRY [R: Purine base, A or G; Y: Pyrimidine base, C or U] sequence motif) were screened as previously reported [54]. Further confirmation of the stem-loop structures was conducted using the whole mRNA sequence in the RNAfold platform by the minimum free energy method. Secondary structure of 3’UTR of the mRNA of interest was visualized with the Forna program, and alignment of 3’UTR of multiple species was performed and visualized using Clustal Omega [55].

### Luciferase reporter assay

The luciferase reporter assay to assess Regnase-1 targeted transcripts was performed as previously described [37]. Briefly, different fragments of the 3’ UTR of *Bcl2l1* transcript (ENSMUST00000109820) were amplified with PCR using specific primers and inserted into the pmirGLO luciferase reporter plasmid (Promega, E1330). For the expression plasmids, the corresponding cDNA fragments of wild-type, the MALT1-resistant Regnase-1 mutant R111A, and the nuclease-dead D141N mutant were cloned into the pMYs-IRES-GFP backbone to generate the Reg1, Reg1-R111A, and Reg1-D141N expression plasmids, respectively. HEK293 cells (ATCC authenticated, Manassas, VA) were co-transfected with pmirGLO-3’UTR plasmid or pmirGLO-empty plasmid together with Reg1, Reg1-R111A, or Reg1-D141N expression plasmid or empty control plasmid by Lipofectamine 3000. After 24 h, cells were lysed, and luciferase activity was determined using the Dual-Luciferase Reporter Assay system (Promega, E1910) in a Glomax 20/20 Luminometer (Promega). The gene encoding Renilla luciferase on the pmirGLO plasmid served as an internal control.

### mRNA stability assay

FACS-sorted WT and R111A CD4^+^ T cells were stimulated with anti-CD3 (5 µg/mL) and soluble anti-CD28 (1 µg/mL) at a density of 5 × 10^5^ cells/mL for 6 h. Then, the CD4^+^ T cells were treated with actinomycin D (5 µg/mL, Sigma, A9415) to stop transcription and collected at 1, 2 and 4 h post-treatment. Cells without actinomycin D treatment served as the 0 h control. Cells collected at the indicated time were subjected to RNA extraction and quantification as described above. The mRNA half-life was calculated and plotted using a nonlinear regression curve fitting method as previously described [56].

### Expansion of Tregs in vivo by IL-2/IL-2Ab complex

IL-2/anti-IL-2 antibody complex (IL-2/IL-2Ab) was prepared by mixing anti-IL-2 mAb (clone JES6-1A12, Invitrogen, 14-7022-81) with mouse IL-2 (PeproTech, 212-12) at a 2:1 molar ratio (5 µg of anti-IL-2 mAb and 1 µg of IL-2) and incubated at 37 °C for 30 min as previously reported [26]. For in vivo Treg expansion, 7-day-old male *LSL-R111A*^*fl/fl*^ and *Foxp3*^*YFP-Cre*^*LSL-R111A*^*fl/fl*^ mice were injected with the IL-2/IL-2Ab complex i.p. (6 µg in 200 µl PBS) or rat IgG2a isotype control for 4 consecutive days. Foxp3^+^ Tregs in the spleen of treated mice were analyzed thereafter by flow cytometry.

### Retroviral-mediated Bcl-xL overexpression in T cells

The cDNA fragment encoding *Bcl2l1* was amplified by PCR and further cloned into the pMYs-IRES-mCherry, a retroviral vector modified by replacing GFP with mCherry based on Cell Biolabs’ pMYs-IRES-GFP backbone, and this pMYs-IRES-mCherry vector served as an empty control vector. Retroviral particles were produced by transfecting Plat-E cells as previously described [24]. Naïve CD4^+^ T cells isolated from *R26*^*CreERT2*^*R111A*^*fl/+*^ mice were stimulated with anti-CD3 (1.5 µg/mL) plus APCs for 18 h and incubated with freshly prepared retroviral particles as previously reported. The transduced CD4^+^ T cells were further cultured for 1 day and then harvested to label with CTV. The transduced and CTV-labeled cells were adoptively transferred into *Rag1*^*–/–*^ mice, followed by 3 doses of tamoxifen treatment (75 mg/kg, i.p.), and T cell (Tconv and Treg) expansion and survival in vivo were assessed 7 days later.

### Statistical analysis

Data are presented as mean ± SD. Sample size was chosen based on our published data sets, and statistical analyses were performed using Prism 10 (GraphPad software). The log-rank test was used for survival analysis. A 2-tailed, unpaired Student’s t test, one-way ANOVA with multiple comparisons or two-way ANOVA with multiple comparisons was used to generate *P* values in comparison among groups, as specified in the corresponding figure legends. *P* < 0.05 was considered statistically significant. The sample sizes are provided in the figure legends to indicate independent replicates used for statistical analyses.

## Supplementary information

Supplemental figures and legends

Reproducibility checklist

Western blot unprocessed images

## Data Availability

RNA-seq data and HyperTRIBE-seq data for this project have been deposited at NCBI’s Gene Expression Omnibus (GEO) under the following accession numbers: GSE325328, GSE325407.
