# Supplementary material for: Genetically modulating the RNA-binding protein Regnase-1 reveals its critical role in regulatory T cell homeostasis and function in vivo
Source: Cell Death Dis. 2026 Jul 1;17(1):814. doi: 10.1038/s41419-026-09055-8 (PMC13586167; doi:10.1038/s41419-026-09055-8)
Supplement: Supplementary file 1 [file 41419_2026_9055_MOESM1_ESM.pdf]

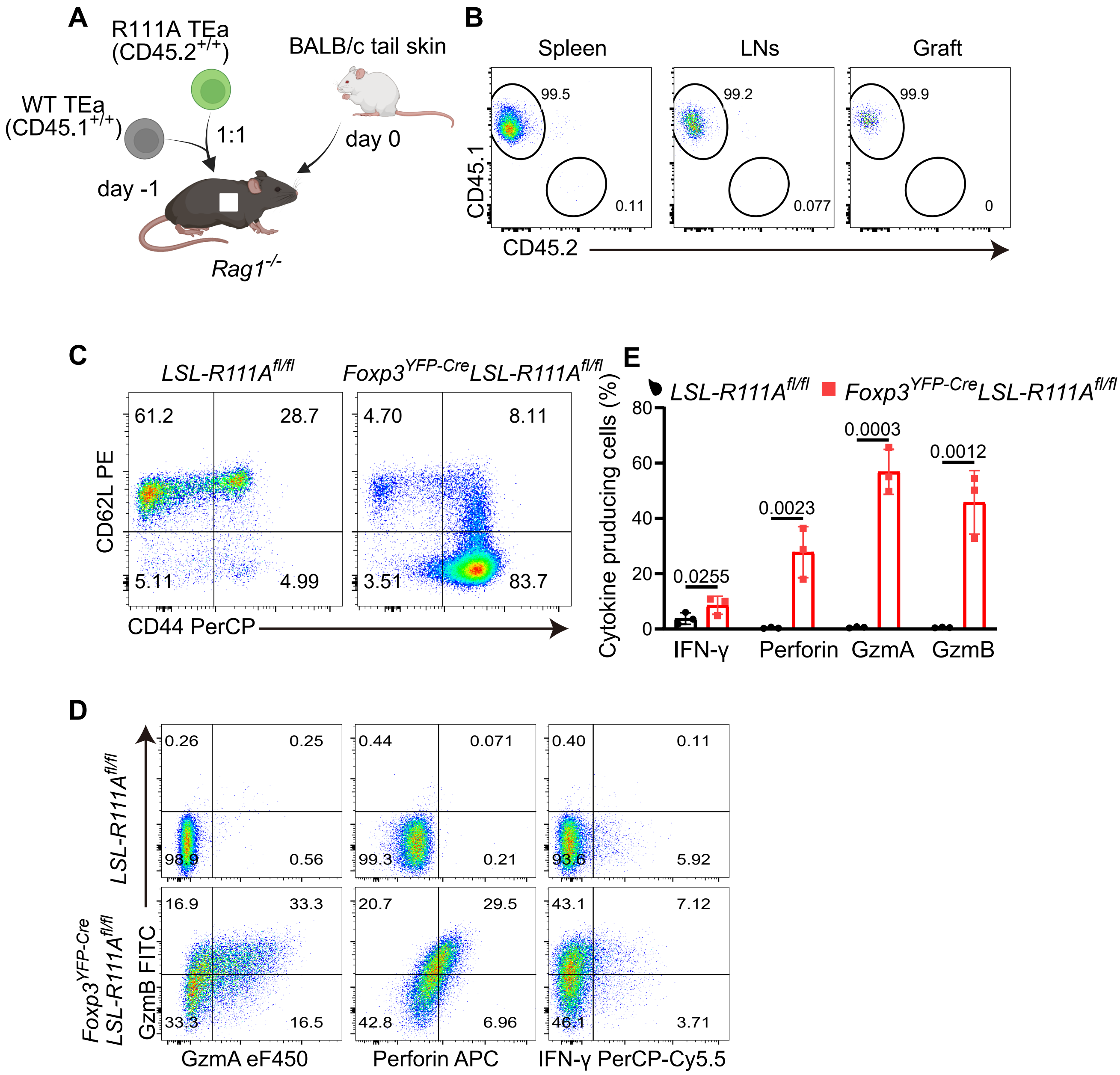
A
R111A TEa
(CD45.2+/+)
WT TEa
(CD45.1+/+)
1:1
BALB/c tail skin
day 0
day -1
Rag1-/-
B
Spleen
LNs
Graft
99.5
0.11
99.2
0.077
99.9
0
CD45.1
CD45.2
C
LSL-R111Afl/fl
Foxp3YFP-CreLSL-R111Afl/fl
61.2
28.7
5.11
4.99
4.70
8.11
3.51
83.7
CD62L PE
CD44 PerCP
E
LSL-R111Afl/fl
Foxp3YFP-CreLSL-R111Afl/fl
Cytokine prducing cells (%)
80
60
40
20
0
0.0255
0.0023
0.0003
0.0012
IFN-γ
Perforin
GzmA
GzmB
D
LSL-R111Afl/fl
Foxp3YFP-Cre LSL-R111Afl/fl
GzmB FITC
0.26
0.25
98.9
0.56
0.44
0.071
99.3
0.21
0.40
0.11
93.6
5.92
16.9
33.3
33.3
16.5
20.7
29.5
42.8
6.96
43.1
7.12
46.1
3.71
GzmA eF450
Perforin APC
IFN-γ PerCP-Cy5.5

Supplemental figure legends

Supplemental figure 1. The survival defect of the R111A-expressing T cells and the effector phenotypes of $CD8^+$ T cells from the autoimmune male *$Foxp3^{YFP\text{-}Cre}$LSL-$R111A^{fl/fl}$* mice

(A) Experimental design showing co-transfer of sorted WT TEa and R111A TEa cells into *$Rag1^{-/-}$* hosts grafted with the MHC-mismatched BALB/c skin grafts. WT and R111A TEa cells were marked by the CD45.1/2 congenic markers. The transferred T cells in the *$Rag1^{-/-}$* hosts were analyzed 21 days after skin transplant in the same hosts.

(B) Flow cytometry plots showing WT TEa ($CD45.1^+$) and R111A TEa ($CD45.2^+$) in the spleen, lymph nodes, and the skin grafts from *$Rag1^{-/-}$* hosts transplanted with BALB/c skin grafts.

(C) Representative flow cytometry plots showing $CD44^{hi}CD62L^-$ T cells in the CD8+ population from *LSL-$R111A^{fl/fl}$* and *$Foxp3^{Cre}$LSL-$R111A^{fl/fl}$* mice.

(D-E) Representative flow cytometry plots (D) and relative percentages (E) of cytokine-producing splenic $CD8^+$ T cells from male *LSL-$R111A^{fl/fl}$* and *$Foxp3^{YFP\text{-}Cre}$LSL-$R111A^{fl/fl}$* mice (n = 3 mice per group).

Data are presented as mean ± SD. Results are representative of at least three independent experiments (B-E). *P* values are determined by two-tailed unpaired Student's *t* test (E).

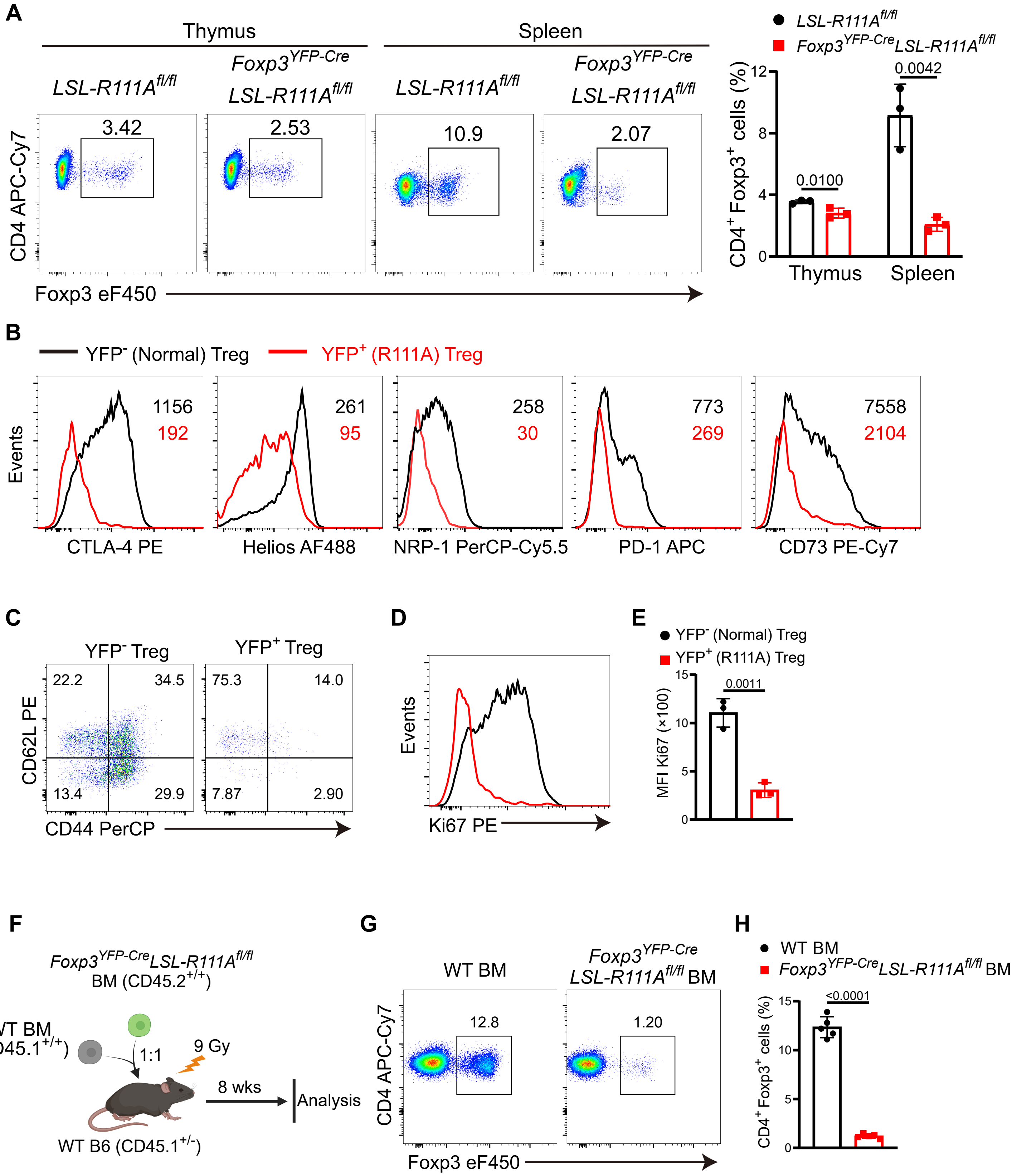

A
Thymus
Spleen
LSL-R111A fl/fl
Foxp3 YFP-Cre LSL-R111A fl/fl
3.42
2.53
10.9
2.07
CD4 APC-Cy7
Foxp3 eF450
LSL-R111A fl/fl
Foxp3 YFP-Cre LSL-R111A fl/fl
0.0100
0.0042
CD4+ Foxp3+ cells (%)
Thymus
Spleen
B
YFP- (Normal) Treg
YFP+ (R111A) Treg
Events
1156
192
261
95
258
30
773
269
7558
2104
CTLA-4 PE
Helios AF488
NRP-1 PerCP-Cy5.5
PD-1 APC
CD73 PE-Cy7
C
YFP- Treg
YFP+ Treg
22.2
34.5
13.4
29.9
75.3
14.0
7.87
2.90
CD62L PE
CD44 PerCP
D
Events
Ki67 PE
E
YFP- (Normal) Treg
YFP+ (R111A) Treg
0.0011
MFI Ki67 (×100)
F
Foxp3 YFP-Cre LSL-R111A fl/fl BM (CD45.2+/+)
WT BM (CD45.1+/+)
1:1
9 Gy
8 wks
Analysis
WT B6 (CD45.1+/-)
G
WT BM
Foxp3 YFP-Cre LSL-R111A fl/fl BM
12.8
1.20
CD4 APC-Cy7
Foxp3 eF450
H
WT BM
Foxp3 YFP-Cre LSL-R111A fl/fl BM
<0.0001
CD4+ Foxp3+ cells (%)

Supplemental figure legends

Supplemental figure 2. Foxp3$^+$ Tregs expressing the R111A mutant show intrinsic survival defects in the periphery

(A) Flow cytometry plots (left) showing Foxp3$^+$CD4$^+$ Tregs in the thymus versus spleen of 7-day-old male *LSL-R111A*$^{fl/fl}$ and *Foxp3*$^{YFP\text{-}Cre}$*LSL-R111A*$^{fl/fl}$ mice. Bar graphs (right) showing the relative percentage of Foxp3$^+$ Tregs in CD4$^+$ T cells in thymus and spleen (n = 3 mice).

(B) Representative flow cytometry plots showing expression of Treg-associated suppressive molecules (CTLA-4, Helios, NRP-1, PD-1 and CD73) on YFP$^+$ and YFP$^–$ Tregs in the spleen of female *Foxp3*$^{YFP\text{-}Cre/+}$*LSL-R111A*$^{fl/fl}$ mice.

(C) Flow cytometry plots showing CD44^{hi}CD62L$^–$ in YFP$^+$ and YFP$^–$ splenic Treg populations from female *Foxp3*$^{YFP\text{-}Cre/+}$*LSL-R111A*$^{fl/fl}$ mice.

(D-E) Representative histograms (D) and bar graphs (E) showing Ki67 expression in YFP$^+$ and YFP$^-$ Tregs in spleen of female *Foxp3*$^{YFP\text{-}Cre/+}$*LSL-R111A*$^{fl/f}$ mice (n = 3 mice).

(F) A schematic diagram showing the generation of bone marrow (BM) chimeric mice. Lethally irradiated WT B6 CD45.1$^{+/-}$ mice were reconstituted with a 1:1 mixture (5 million cells each) of 3-week-old male CD45.1$^+$ WT and CD45.2$^+$ *Foxp3*$^{YFP\text{-}Cre}$*LSL-R111A*$^{fl/fl}$ BM cells, followed by flow cytometry analysis 8 weeks later.

(G-H) Representative flow cytometry plots (G) and relative percentages (H) of CD4$^+$Foxp3$^+$ Tregs derived from the transferred WT or *Foxp3*$^{YFP\text{-}Cre}$*LSL-R111A*$^{fl/fl}$ BM cells (n = 3 mice).

Data are presented as mean ± SD. Results are representative of three independent experiments (A-E) or pooled from 2 independent experiments (H). Statistical significance was determined by two-tailed unpaired Student's *t* test (E, H).

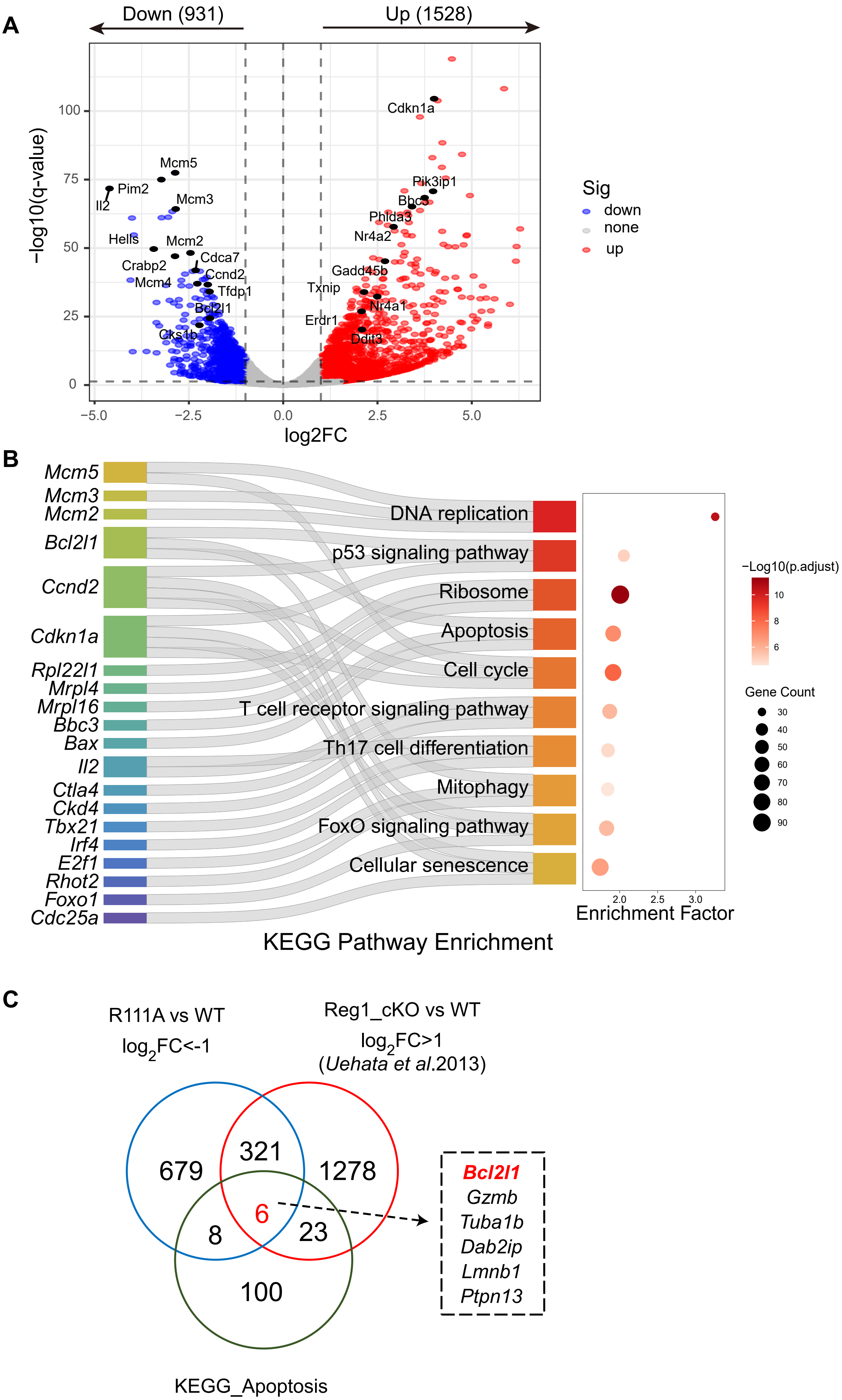
A
Down (931)
Up (1528)
−log10(q-value)
log2FC
Cdkn1a
Pik3ip1
Bbc3
Phlda3
Nr4a2
Gadd45b
Txnip
Nr4a1
Erdr1
Ddit3
Mcm5
Pim2
Il2
Mcm3
Hells
Mcm2
Cdca7
Crabp2
Ccnd2
Mcm4
Tfdp1
Bcl2l1
Cks1b
Sig
down
none
up
B
Mcm5
Mcm3
Mcm2
Bcl2l1
Ccnd2
Cdkn1a
Rpl22l1
Mrpl4
Mrpl16
Bbc3
Bax
Il2
Ctla4
Ckd4
Tbx21
Irf4
E2f1
Rhot2
Foxo1
Cdc25a
DNA replication
p53 signaling pathway
Ribosome
Apoptosis
Cell cycle
T cell receptor signaling pathway
Th17 cell differentiation
Mitophagy
FoxO signaling pathway
Cellular senescence
Enrichment Factor
−Log10(p.adjust)
Gene Count
KEGG Pathway Enrichment
C
R111A vs WT
log2FC<-1
Reg1_cKO vs WT
log2FC>1
(Uehata et al.2013)
679
321
1278
6
8
23
100
KEGG_Apoptosis
Bcl2l1
Gzmb
Tuba1b
Dab2ip
Lmnb1
Ptpn13

Supplemental figure legends

Supplemental figure 3. Transcriptomic analysis of WT and R111A-expressig T cells revealing impaired expression of *Bcl2l1* mRNA.

(A) A volcano plot showing the transcriptional profiles of $CD4^+$ T cells expressing the R111A mutant following anti-CD3/anti-CD28 stimulation for 12 h. The differentially expressed genes ($Log_2FC > 1$, q-value < 0.05) were indicated by blue and red dots on the plot, respectively. Genes related to DNA replication, cell cycle, apoptosis, and T cell survival were annotated.

(B) Bubble plot combined with Sankey diagram demonstrating pathway enrichment analysis of differentially expressed genes of R111A-expressing $CD4^+$ T cells based on the KEGG database. Top 10 statistically significant KEGG pathways, including apoptosis and the most differentiated *Bcl2l1* gene within this pathway, were shown.

(C) Venn diagram showing the genes shared among the down-regulated genes in R111A $CD4^+$ T cells, up-regulated genes in Regnase-1 KO $CD4^+$ T cells, and genes associated with apoptosis pathways in KEGG database. The *Bcl2l1* gene (highlight in red) was ranked as one of the most affected genes among the shared genes.

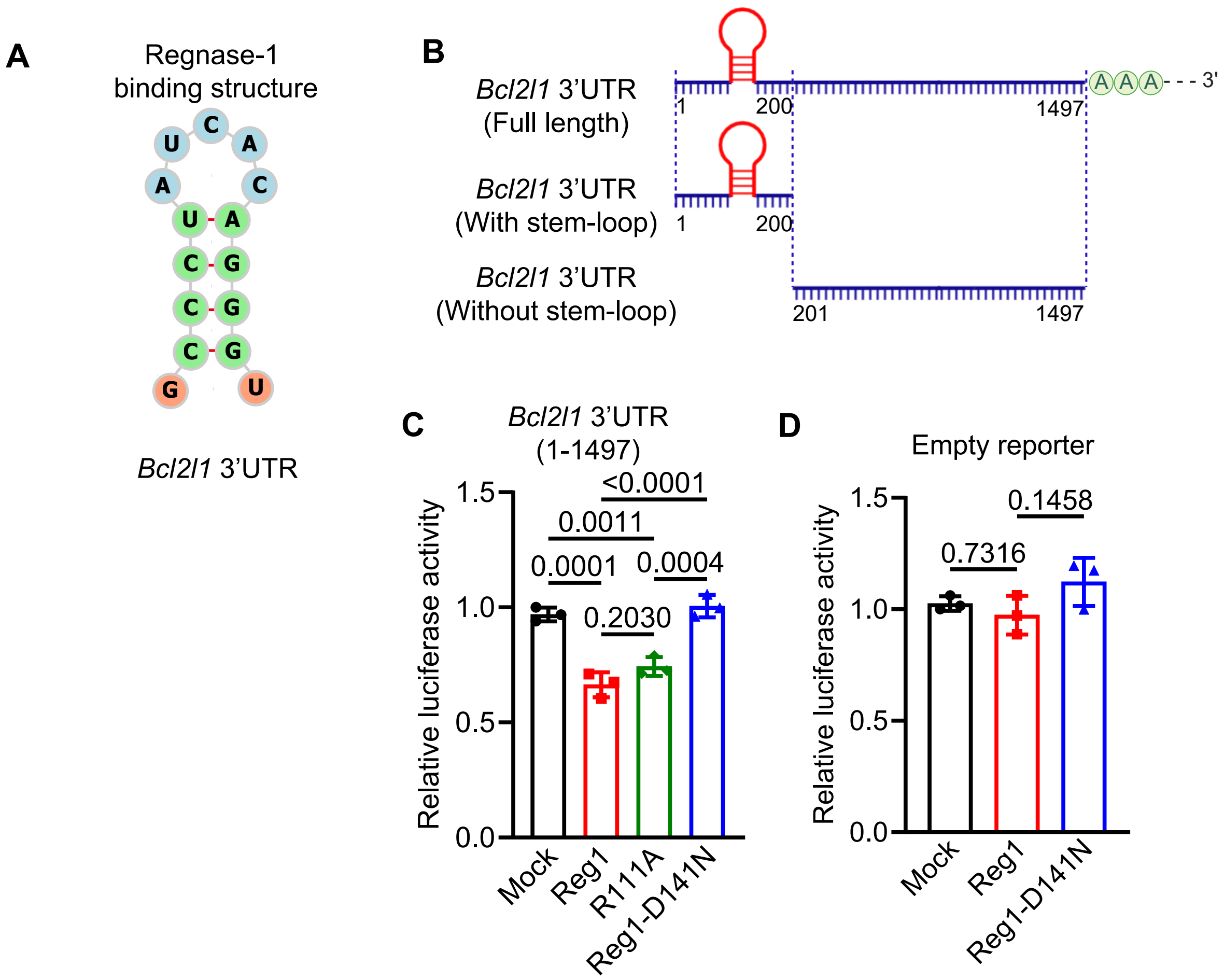
A
Regnase-1
binding structure
U C A A C U A C G C G C G G U
Bcl2l1 3'UTR
B
Bcl2l1 3'UTR
(Full length)
1
200
1497
AAA - - - 3'
Bcl2l1 3'UTR
(With stem-loop)
1
200
Bcl2l1 3'UTR
(Without stem-loop)
201
1497
C
Bcl2l1 3'UTR
(1-1497)
Relative luciferase activity
1.5
1.0
0.5
0.0
<0.0001
0.0011
0.0001
0.0004
0.2030
Mock
Reg1
R111A
Reg1-D141N
D
Empty reporter
Relative luciferase activity
1.5
1.0
0.5
0.0
0.1458
0.7316
Mock
Reg1
Reg1-D141N

Supplemental figure legends

Supplemental figure 4. The stem-loop structure at the 3'UTR region of *Bcl2l1* mRNA is targeted by Regnase-1.

(A) A schematic diagram showing sequences targeted by Regnase-1 at the stem-loop structure at the 3' UTR region of *Bcl2l1* mRNA. Stem-loop structure was predicted with the RNAfold software.

(B) A schematic diagram showing different lengths of 3'UTR of *Bcl2l1* (with or without specific stem-loop structure) that were cloned into the luciferase pmirGLO reporter plasmids.

(C) Luciferase reporter activities in HEK293 cells transfected with pmirGLO reporter plasmid containing full length of 3'UTR of mouse *Bcl2l1*, together with expression plasmids for wild-type (Reg1), the R111A mutant, the nuclease-dead Reg1-D141N mutant, or empty vector control (Mock), indicating that R111A plasmid shared the similar function of Reg1 expression plasmid (n = 3 independent replicates).

(D) Luciferase reporter activities in HEK293 cells transfected with empty pmirGLO reporter plasmid and different expression plasmids of Regnase-1 (Mock, Reg1 and Reg1-D141N), confirming that the reduced luciferase reporter activities relied on the reporter plasmid containing the Regnase-1 targeted sequences (n = 3 independent replicates).

Data are presented as mean ± SD. Results are representative of three independent experiments (C-D). Statistical significance was determined by one-way ANOVA with Tukey's multiple comparison test (C-D).
