# Supplementary material for: Genetically modulating the RNA-binding protein Regnase-1 reveals its critical role in regulatory T cell homeostasis and function in vivo
Source: Cell Death Dis. 2026 Jul 1;17(1):814. doi: 10.1038/s41419-026-09055-8 (PMC13586167; doi:10.1038/s41419-026-09055-8)
Supplement: Supplementary file 3 [file 41419_2026_9055_MOESM3_ESM.pdf]

Figure 1D

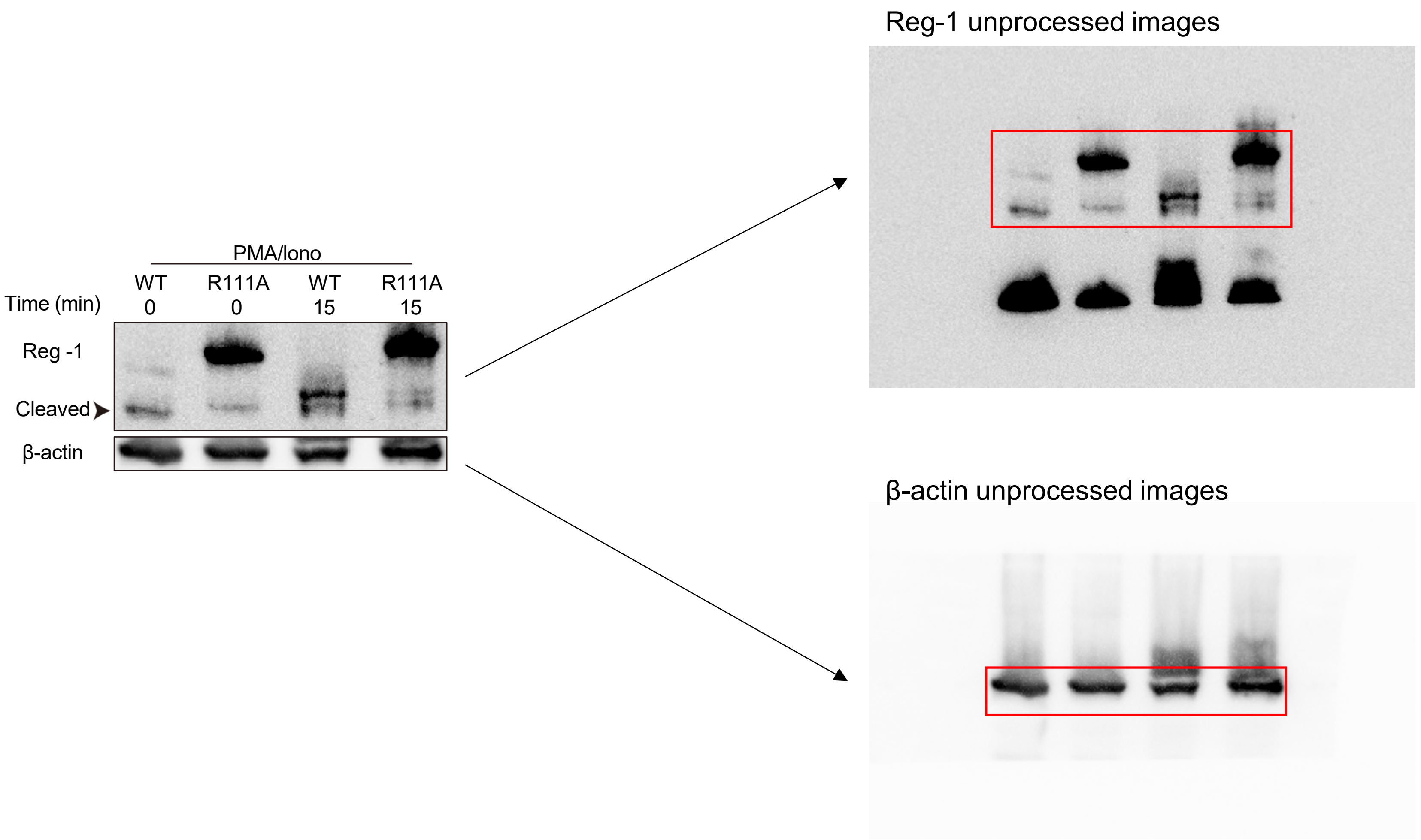
PMA/Iono
WT
R111A
WT
R111A
Time (min)
0
0
15
15
Reg -1
Cleaved
β-actin
Reg-1 unprocessed images
β-actin unprocessed images

Figure 5F

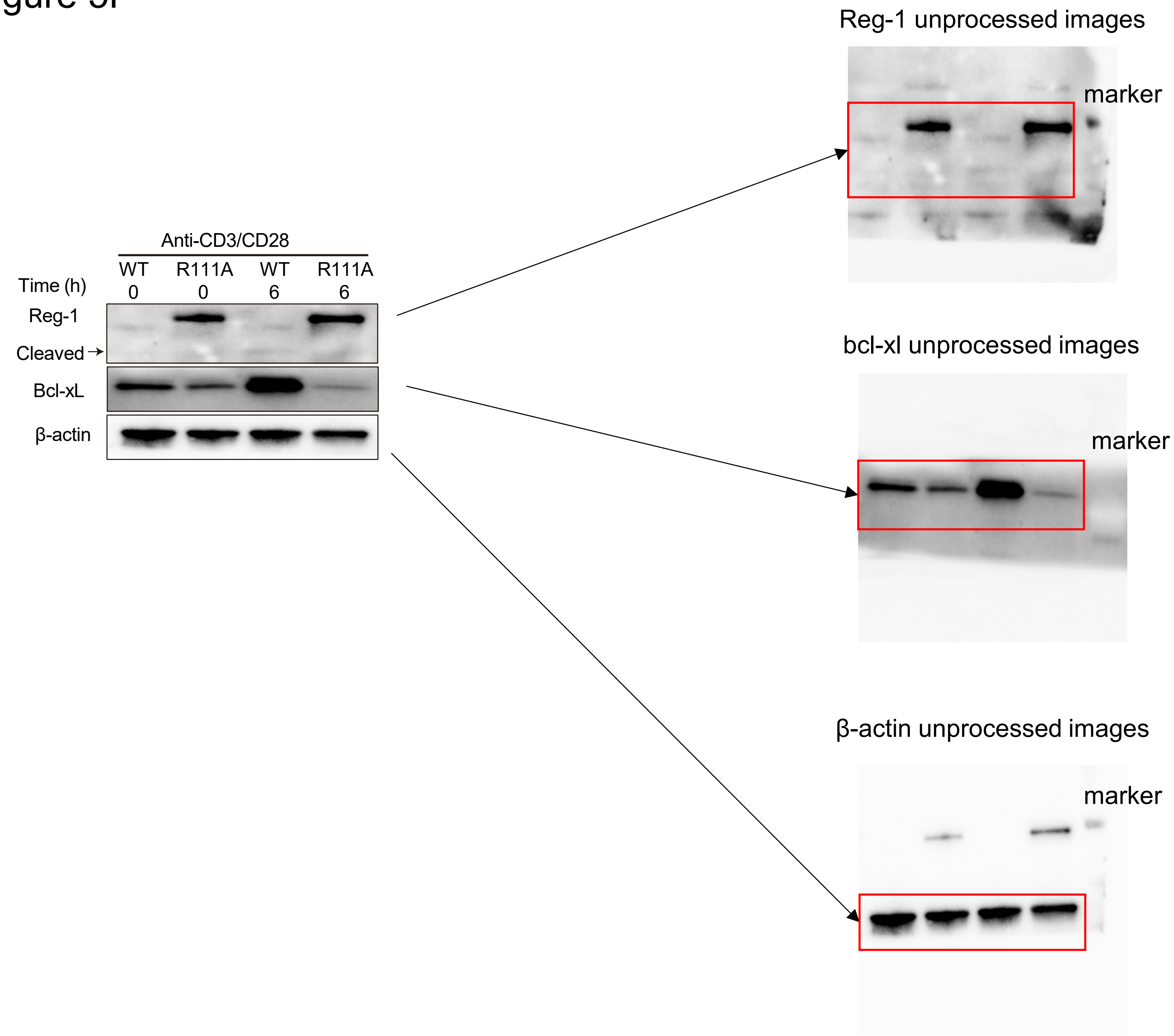

Anti-CD3/CD28
Time (h)
WT 0
R111A 0
WT 6
R111A 6
Reg-1
Cleaved
Bcl-xL
β-actin
Reg-1 unprocessed images
marker
bcl-xl unprocessed images
marker
β-actin unprocessed images
marker
